# Effects of isolated, confined and extreme environments on parameters of the immune system - a systematic review

**DOI:** 10.3389/fimmu.2025.1532103

**Published:** 2025-03-25

**Authors:** Bea Klos, Alina Kaul, Emily Straube, Verena Steinhauser, Celina Gödel, Franziska Schäfer, Claude Lambert, Paul Enck, Isabelle Mack

**Affiliations:** ^1^ Internal Medicine VI, University Hospital Tübingen, Tübingen, Germany; ^2^ Cytometry Unit, Immunology Laboratory, Saint-Etienne University Hospital, Saint-Étienne, Lyon, France; ^3^ LCOMS/ENOSIS Environmental Toxicology, University of Lorraine, Metz, France

**Keywords:** immune system, ICE environments, antigen-limited environments, immune dysregulation, antigen diversity, space analogues, resilience, bed rest

## Abstract

**Background:**

The immune system is a crucial part of the body’s defense against infection and disease. However, individuals in antigen-limited environments face unique challenges that can weaken their immune systems. This systematic review aimed to investigate the impact of an exposure to an isolated, confined and extreme (ICE) environment with limited antigen diversity on human immune parameters.

**Methods:**

A systematic literature search was conducted using PubMed, Web of Science and Cochrane Library to identify relevant studies on immune system parameters in ICE environments. The studies were grouped by ICE type (space missions, microgravity simulations like bed rest studies, space simulation units like MARS500, and Antarctic research stations) to allow for clearer comparison and analysis of immune outcomes.

**Results:**

Analysis of 140 studies revealed considerable heterogeneity in study designs and outcomes, reflecting the complexity of immune responses across ICE environments. Nevertheless, immune dysregulation was consistently observed across environments. Space missions and Antarctic stations, in particular, showed pronounced immune changes, likely due to low antigen diversity and extreme conditions, with higher rates of infections and allergic responses suggesting increased vulnerability. Space simulation units exhibited immune changes similar to those in actual space missions, while gravity simulation studies, which focus on fluid shifts and bone loss, showed fewer immune alterations. Across environments, most immunological measures returned to baseline after isolation, indicating resilience and the potential for recovery upon re-exposure to diverse antigens.

**Conclusion:**

Reduced antigen diversity in ICE environments disrupts immune function, with effects often compounded by extreme conditions. Although immune resilience and recovery post-isolation are promising, the heterogeneity in current studies highlights the need for targeted research to identify specific immune vulnerabilities and to develop countermeasures. Such measures could reduce immune-related health risks for individuals in isolated settings, including astronauts, polar researchers, and vulnerable populations on Earth, such as the elderly or immunocompromised, thereby enhancing resilience in confined environments.

**Systematic Review Registration:**

https://www.crd.york.ac.uk/prospero/, identifier CRD42023476132.

## 1 Introduction

The precision of the immune system in differentiating between self and non-self, as well as between pathogens and saprophytes, is crucial for sustaining health (1). This ability not only ensures effective defense against harmful agents but also supports other vital functions of the immune system. Beyond direct defense, the immune system is crucial for facilitating wound healing, monitoring cellular changes, maintaining homeostasis within the body, and tolerating food and airborne particles such as plant matter and animal dander. Disruptions in these functions can lead to severe infections, tumor emergence, autoimmune disorders, or allergies (1).

The human immune system is shaped by a complex interplay of internal factors like genetic (2) and hormonal regulation (3, 4), health conditions such as obesity (5) and clinical depression (6), along with antigen exposure from external sources like pollution, nutrition, stress, and infections (7). There is substantial evidence demonstrating that continuous interaction between the immune system and antigen exposure is crucial for maintaining immune fitness, discerning pathogens, and mounting an effective defense (1). Furthermore, research shows that continuous and diverse antigenic stimulation strengthens immune function (8, 9) by ‘educating’ (10) the immune system to effectively distinguish pathogens from benign substances. In the absence of regular stimulation, immune memory may deteriorate, leading to “immune amnesia,” wherein the immune system fails to properly recognize or respond to previously encountered antigens. Therefore, in immunocompromised states—such as sepsis, toxic shock syndrome, cytokine storms, or prolonged idleness—consistent environmental exposure, including microbial interactions and a diverse diet, is essential for preserving immune function (11).

Prolonged exposure to antigen-limited environments raises concerns about potential immune impairments, increasing susceptibility to infections and immune dysregulation (12). Indeed, research shows an increased risk of allergies, chronic inflammation, and autoimmune disorders in communities with lower antigen exposure (13–16). Furthermore, children born and raised in highly sanitized environments exhibit higher incidences of allergies and autoimmune conditions compared to those from more natural settings, particularly in early childhood when the immune system is most adaptive to environmental inputs (14, 16). In adulthood, these effects are less pronounced but still evident, as seen in migrants from developing to industrialized nations, where allergy rates increase gradually over time, indicating slower immune adaptation (17–19).

Comparable effects on immune function are observed in isolated, confined, and extreme (ICE) environments, such as space stations, polar research stations, and long-duration exploration missions (12, 20, 21). These settings often feature unique extreme environmental stressors incompatible with animal, microbial, or plant life, resulting in limited antigenic diversity. The combination of isolation, these stressors, and limited access to medical care makes immune function particularly vulnerable, emphasizing the need to understand these factors for safeguarding health in challenging environments.

Space exemplifies an ICE environment, where immune system dysregulation is well-documented (21, 22), affecting immunological parameters such as leukocyte distribution, cellular function, and cytokine production profiles (22). Clinical reports from space missions have documented adverse outcomes, including rashes and persistent rhinitis (23). However, in addition to antigen-limitation, astronauts are exposed to radiation, microgravity, fluid shifts, and circadian rhythm disruptions - factors that possibly exacerbate immune dysregulation.

As ICE environments, spaceflight analogues such as Antarctic expeditions, prolonged bed rest studies, and other space simulation programs (e.g., MARS-500 or SIRIUS), reflect key physiological and psychological effects observed in spaceflight (12), though they cannot fully replicate all conditions of true space missions (24). Insights from Antarctic studies revealed that individuals experience reduced immune responsiveness, including T cell dysfunction, decreased proliferation, and diminished responses in skin-based immunity tests (25). These impairments were evidenced in a study conducted at an Antarctic research station, which reported lowered salivary immunoglobulin (Ig) levels, suggesting that isolation impacts mucosal immunity (26). Japanese and Indian studies in Antarctica corroborate these findings, demonstrating alterations in leukocyte counts and weakened antibody production (27–30). In a 42-day head-down tilt bed rest study (31), designed to mimic the effects of microgravity, volunteers experienced shifts in immune cell populations, with an increase in polymorphonuclear cells and stable levels of T lymphocytes and monocytes. Cytokine secretion patterns were altered, particularly with elevated levels of Interleukin (IL)-1β, indicating a pro-inflammatory response, while stress hormone cortisol levels slightly decreased. Latent viral reactivation, including Epstein-Barr virus (EBV) and *Varicella zoster* virus (VZV), further highlighted the immune system’s vulnerability during prolonged bed rest (32). Although immune alterations are evident, bed rest studies primarily focus on simulating microgravity conditions, including fluid shifts, bone loss, and muscle deterioration, rather than replicating the degree of antigen limitation seen in space or Antarctic research (24). However, the precise contribution of limited antigenic diversity to these alterations remains unclear, as ICE environments present a highly complex and multifactorial setting. Besides antigen diversity, additional environmental factors – such as radiation exposure (33) or high altitude (33) - often co-occur with physiological (24) or psychological stressors (25), making it challenging to isolate their individual effects on immune responses. Depending on the research focus, different ICE environments offer unique advantages.

Prolonged exposure to ICE environments and their associated stressors may induce dysregulated immune responses, potentially linked to adverse health outcomes (24). Although recent reports provide evidence that individuals returning from extended stays in ICE environments often acquire infections or develop new allergies (24, 34, 35), the link between ICE-induced immune dysregulation and clinical outcomes remains largely unexplored. Moreover, there are critical gaps in understanding how prolonged exposure to ICE environments impacts immune function, particularly in the context of stressors such as microgravity, increased radiation, hypoxia, limited antigenic diversity and psychological stress. It remains unclear whether immune alterations stem primarily from the isolation itself, from environmental or psychological stressors, or from a combination of both factors. Clarifying this requires comparing immune responses across different ICE habitats, each with distinct combinations of isolation and environmental stressors. Currently, no comprehensive review has systematically investigated immune dynamics during prolonged exposure to these environments. Addressing these knowledge gaps will not only enhance our understanding of immune function in ICE environments but also provide critical insights into the potential health risks posed by overly sanitized urban settings, informing future research and preventive strategies. Given the current gaps in knowledge, the following research questions are proposed:

1. Does long-term isolation (>28 days) cause changes in immune system regulation, irrespective of the ICE environment or duration?
2. Does immune cell function fully recover to pre-isolation levels after prolonged isolation (>28 days), regardless of the specific ICE environment or duration?

## 2 Methods

### 2.1 Databases and search strategy

This systematic literature research adheres to the Preferred Reporting Items for Systematic Reviews and Meta-Analyses (PRISMA) guidelines (36). To identify all relevant studies examining the impact of ICE environments on the immune system the databases PubMed, Web of Science and Cochrane Library (Wiley) were searched on 22^nd^ of November 2022, with an update on January 28, 2025. The protocol of this systematic review is registered on the PROSPERO platform with the registration number CRD42023476132. The full search strategy was conducted in assistance with a specialized librarian and is documented in the **Supplementary Text S1** in **Supplementary Data Sheet 1**. It is composed of 3 modules: ICE conditions, human immune system and exclusion of animals. A highly specific search term was chosen to accurately represent isolation conditions, drawing on successful terms used in a previous literature search (37).

### 2.2 Eligibility criteria

Inclusion criteria were based on the five PICOS dimensions, i.e., participants, intervention, comparator, outcome and study design (38).

Population: Healthy adults regardless of sex, age, or weight status, who had been in an ICE environment for at least four weeks (28 days) were included. A 28-day minimum threshold was set to exclude shorter isolation periods, such as those experienced during COVID-19 lockdowns, to maintain focus on the isolation effects relevant to space missions and research station environments. Studies involving animals were systematically excluded, as this review focuses exclusively on human immune responses.

Intervention: Isolation in environments with uniform or monotone/reduced antigen exposure. Such conditions are found a) in space missions, b) in space analogues such as MARS-500, SIRIUS and Lunar Palace-1, bed rest studies or extreme environmental locations such as expeditions in Antarctica, the Arctic and Siberia. Additionally, other environments that still share the key characteristics of ICE conditions are considered, including maritime expeditions, sledding excursions, or prolonged swimming expeditions.

Comparison: Studies with control groups were allowed but not necessary.

Outcome: Assessment of *in vivo* parameters of the human immune system without additional *in vitro* stimulation. Studies relying exclusively on subjective symptom reports, without biomarker assessment related to the immune system, were excluded.

Study design: Randomized controlled trials or non-randomized controlled trials with any publication date and written in English, German or Russian. Only original articles were included.

### 2.3 Screening and literature organization

To identify eligible studies, the search results of the databases were combined, and the duplicates were removed. Two authors (BK, AK) independently screened titles and abstracts to identify relevant trials. Full-text articles were evaluated regarding their eligibility (BK, AK), with uncertainties being discussed between the authors (<3% cases). A third author (IM) was involved if discrepancies persisted.

To reduce heterogeneity, the studies were categorized into four groups based on ICE conditions:

1. **Space missions:** Isolation/confinement experienced by astronauts during spaceflights, such as missions aboard the International Space Station (ISS) or similar space missions.
   Extreme conditions: microgravity, high levels of cosmic radiation, and disruptions of the circadian rhythm, isolation from civilization resulting in antigen-limitation.
2. **Gravity simulation studies (space analogue):** Isolation/confinement in environments that simulate microgravity conditions, such as bed rest studies with head-down tilt.
   Extreme conditions: altered body fluid distribution, prolonged immobility, the absence of normal gravitational forces, antigen limitation up to a certain degree.
3.** Terrestrial artificial habitats, indoor (space analogue):** Isolation/confinement in environments that simulate space conditions in a specific facility. Examples include MARS-500 or Lunar Palace-1.
   Extreme conditions: artificial atmosphere control, limited space, isolation from civilization resulting in antigen-limitation, and lack of natural light or weather.
4. **Terrestrial natural habitats, outdoor (space analogue):** Isolation/confinement in natural and remote environments. Examples include Arctic or Antarctic research stations.
  Extreme conditions: harsh weather conditions (e.g., extreme cold), low oxygen levels, high radiation, isolation from civilization resulting in antigen-limitation, and continuous darkness or daylight during polar seasons.

Certain experiments may have yielded multiple publications addressing immune-related data. As the outcomes may differ in detail and description, all publications are listed in the tables. However, a summary of these studies is provided in the text and data evaluation sections. In case that studies can be assigned to several groups, they were labelled respectively.

### 2.4 Data items and statistics

The extracted information from each article includes details on study characteristics, isolation conditions, and immunological measures. Study characteristics are presented using original data and summarized in tabular form, including mean, standard deviation, minimum and maximum values for sample size, age, and study duration. Median and interquartile range are provided where applicable.

The primary outcomes focus on evaluating the reports of changes or stabilities in immune markers, with the analysis emphasizing the consistency of reported trends across studies rather than direct immunological measure quantification. Outcomes were analyzed for both isolation periods (pre-/during-comparisons) and recovery periods (during-/post-comparisons), with pre-/post-analyses included when applicable. To improve clarity, consistency, and comparability across immunological measures, study habitats, and time points (pre/during, during/post, pre/post), findings were expressed as the percentage of studies reporting increases, decreases, or stability of immunological measures, rather than using absolute values. For example, instead of reporting specific cytokine concentration changes, we calculated the proportion of studies observing increases, decreases, or stability in cytokine levels. This approach allowed us to highlight general trends across heterogeneous datasets. For the summary, only immune parameters with an average reporting frequency of at least 5%, indicating either variability or stability across all environments, were included. This criterion enhances the reliability of the conclusions by focusing on consistently observed patterns. Parameters showing no changes are not displayed in the summary, but in the **Supplementary Tables S1**-**S4**. Additionally, immunological parameters such as leukocytes, lymphocytes, cytokines, granulocytes and immunoglobulins were sometimes sub-classified in the literature, but not consistently across studies. For example, some studies reported leukocytes with detailed subclassifications (e.g., neutrophils, lymphocytes, monocytes), while others only provided total leukocyte counts. In cases where sub-classifications were absent or inconsistent, the broader immunological measure was included in the analysis and discussed without further subclassification. This approach ensured that the variability in reporting practices did not compromise the integrity of the analysis. Control subjects not exposed to isolation or confinement were excluded from further analyses.

Secondary outcomes included clinical parameters assessing the incidence and progression of infections and allergic responses as well as medication use during and following the intervention.

### 2.5 Risk of bias assessment

The assessment of bias for the included studies was conducted using the Risk of Bias In Non-randomized Studies of Interventions (ROBINS-I) tool (39). Since only non-randomized trials (non-RCTs) were considered in this systematic review, the Cochrane tool was chosen. This tool treats each study as an attempt to replicate a hypothetical pragmatic randomized trial and encompasses seven distinct domains addressing potential bias introduction. The first two domains address issues related to confounding and participant selection before the interventions (“baseline”), while the third domain discusses intervention classification. The remaining four domains address issues after the start of interventions: biases due to deviations from intended interventions, missing data, measurement of outcomes, and selection of the reported result.

The bias rating ranged from ‘Low risk’, indicative of high-quality trials, to ‘Moderate’, ‘Serious’, and ‘Critical risk’. However, no study was excluded based on the risk of bias assessment, as the essential nature of the studies made exclusion impractical.

## 3 Results

**Figure 1:**
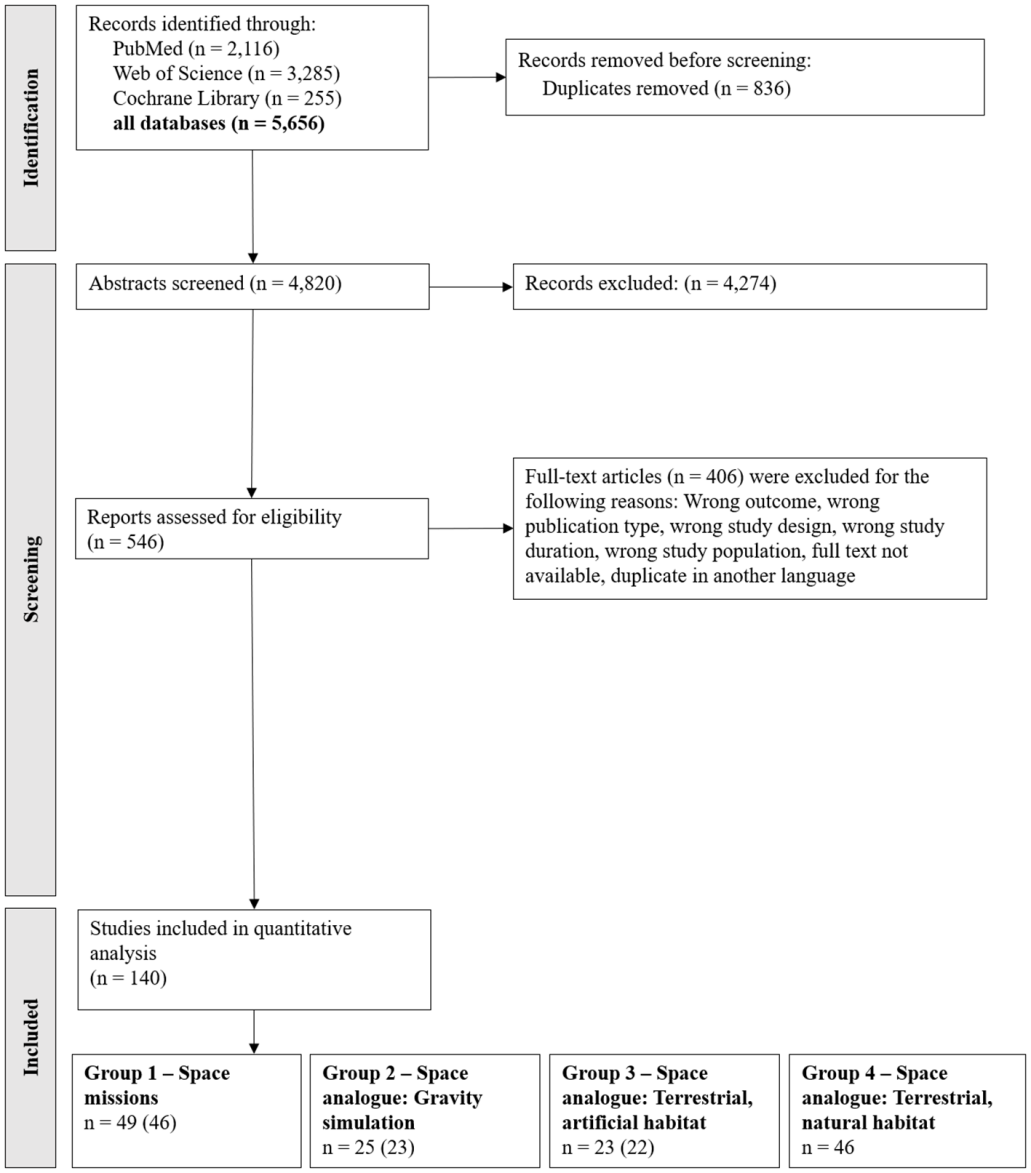
PRISMA Flow chart of the included studies. *Note:* The search was conducted on 22^nd^ of November 2022 (PubMed n = 1,943; Web of Science n = 2,778; Cochrane Library n = 217) and updated on 28^th^ of January 2025 (results in the figure). The search term for the Cochrane Library was adjusted to the updated Cochrane search criteria. However, the search on 28^th^ of January 2025 yielded only 210 studies in total, of which 38 were newly identified compared to the initial search. The included studies were categorized based on their respective habitats. A total of 140 studies were included in the quantitative analysis. Three of these studies covered multiple habitats and are therefore counted in more than one group category. The numbers in parentheses indicate the count after adjusting for these overlaps.

Out of 5,656 identified studies, 140 studies fulfilled criteria for qualitative analysis (**Figure 1**) with 49 studies categorized in group 1 (space studies), 25 studies in group 2 (space analogue: gravity simulations), 23 studies in group 3 (space analogue: terrestrial, artificial habitats) and 46 studies in group 4 (space analogue: terrestrial, natural habitats). Notably, three studies included multiple habitat categories and were therefore included in more than one group.

### 3.1 Study characteristics

The characteristics across the studies are summarized in the text and in **Table 1**. A detailed overview of the characteristics for the single trials is presented in **Table 2**.

**Table 1:** Study characteristics across the studies.

	Group 1 (n = 49): Space missions	Group 2 (n = 25): Gravity simulation studies	Group 3 (n = 23): Terrestrial, artificial habitat	Group 4 (n = 46): Terrestrial, natural habitat
isolation length (d)				
range	49-438	28-370	30-520	30- N.A.
not reported (n)	1	0	1	5
participants (n)				
median (IQR)	8.5 (13.8)	8 (6.3)	6 (3)	16 (14)
range	1-72	2-31	1-26	1-348
not reported (n)	1	2	1	0
age (years)				
range	27-60	20-55	19-67	18-61
not reported (n)	32	10	7	16
sex (f%)				
mean (SD)	12.3 (15.7)	27.3 (44.3)	20.5 (30.1)	8.3 (18.6)
range	0-50	0-100	0-100	0-100
not reported (n)	19	5	9	14

If applicable, study characteristics within each group were reported using median and interquartile range (IQR), as well as mean and standard deviation (SD). Additionally, the range and number of studies not providing the information (n) were noted for each characteristic within each group. d, days; f, female; N.A., not applicable.

**Table 2:** Study characteristics.

ID	Author (y)		origin	isolation type	duration (d)	group	n	age (y)	sex (%f)	sampling
Group 1: Space missions										
1	Agha et al. (2020) (40)		USA	ISS	183-365	–	8	27-53	12.5	blood, saliva
2	Benjamin et al. (2016) (41)		USA	ISS	152-215	–	16	42-55	12.5	blood
3	Berendeeva et al. (42)		RUS	ISS	124-199	–	20	N.A.	N.A.	blood
4	Bigley et al. (43)		USA	ISS	183-365	–	9	45 ± 7	11.1	blood
5	Buchheim et al. (44)		GER	Space	140-181	–	12	41-51	0.0	blood
6	Buchheim et al. (45)		GER	ISS	124-186	–	5	39-54	0.0	blood
7	Buravkova et al. (46)		RUS	ISS	158	–	9	N.A.	N.A.	blood
8	Capri et al. (47)		ITA	ISS	180	–	2	N.A.	0.0	blood
9	Crucian et al. (48)		USA	ISS	177-213	–	8	52 ± 3	0.0	blood
10	Crucian et al. (49)		USA	ISS	180	–	28	49 ± 4	25.0	blood
11	Crucian et al. (50)		USA	ISS (Soyuz)	< 60-180	–	23	53	21.7	blood
12	da Silveira et al. (51)		USA/GBR	Space	120-183	–	59	47 ± 6	20.3	blood
13	Garett-Bakelmann et al. (52)		USA	ISS	340	–	1	50	0.0	blood
14	Bezdan et al. (53)									
15	Gertz et al. (54)									
16	Grigoriev et al. (55)		USSR	MIR (Soyuz)	~ 365	–	2	N.A.	0.0	blood
17	Guseva and Tashpulatov (1979) (56)		USSR	Space	49	–	2	N.A.	0.0	blood, saliva
18	Kimzey (1975, a) (57)		USA	Skylab	84	–	3	N.A.	N.A.	blood
19	Kimzey (1975, b) (58)		USA	Skylab	59	–	3	N.A.	N.A.	blood
20	Kimzey et al. (58)									
21	Konstantinova et al. (59)		USSR	Space	63	–	2	N.A.	0.0	blood
22	Konstantinova et al. (1985) (60)		USSR	Space	75-211	–	14	N.A.	0.0	blood
23	Konstantinova (61)		USSR	Salyut, MIR	N.A.	–	72	N.A.	N.A.	blood
24	Konstantinova et al. (1991) (62)		USSR	Salyut, MIR	112-175	–	16	N.A.	N.A.	blood
					211-355	–	9	N.A.	N.A.	
25	Konstantinova et al. (1995) (63)		RUS	Space	197	–	2	N.A.	N.A.	blood
26	Krieger et al. (64)		USA	ISS	136-290	–	13	38-60	15.4	blood, saliva
27	Kuzichkin et al. (65)		RUS	ISS	115-205	–	15	37-60	0.0	blood
28	Kuzichkin et al. (66)									blood
29	Lesniak et al. (67)		RUS	MIR	130-365	–	24	N.A.	N.A.	
30	Manie et al. (68)		FRA	MIR	151	–	2	N.A.	N.A.	blood
					166	–	2	N.A.	N.A.	
31	Mehta et al. (69)		USA	ISS	277	–	1	N.A.	0.0	blood
32	Mehta et al. (70)		USA	ISS	180	–	1	N.A.	N.A.	blood
33	Meshkov and Rykova (71)		RUS	N.A.	65-366	–	27	N.A.	N.A.	blood
34	Meshkov et al. (72)		RUS	N.A.	179-197	–	8	N.A.	0.0	blood
35	Morukov et al. (73)		RUS	ISS	128-215	–	12	N.A.	N.A.	blood
36	Morukov et al. (74)		RUS	ISS	176-213	–	12	N.A.	N.A.	blood
37	Nikolaeva et al. (1982) (75)		N.A.	N.A.	49	–	47	N.A.	N.A.	blood
38	Pastushkova et al. (2021) (76)		RUS	ISS	169-199	group1	4	44 ± 6	0.0	blood
						group2	3		0.0	
39	Poliakov and Noskov (2005) (77)		RUS	MIR	438	–	N.A.	N.A.	N.A.	blood, saliva
40	Ponomarev et al. (2016) (78)		RUS	ISS	170-199	–	8	32-56	N.A.	blood
41	Rykova et al. (2006) (79)		RUS	ISS	128-195	–	9	N.A.	N.A.	blood
42	Rykova et al. (2008) (80)		RUS	ISS	125-195	–	15	N.A.	N.A.	blood
43	Spielmann et al. (2018) (81)		USA	ISS	180	–	23	52 ± 4	21.7	blood
44	Spielmann et al. (2019) (82)		USA	ISS	180	ISS	23	47 ± 6	13.0	blood
						control	6	33 ± 7	N.A.	
45	Stahn et al. (83)		GER	ISS	180	–	11	50 ± 4	36.4	blood
46	Stowe et al. (2011) (84)		USA	ISS	180	–	18	48 ± 4	16.7	blood
47 48	Vorobyov et al. (1983) (85) Vorob´ev et al. (86)		USSR	Salyut (Soyuz)	75	crew 5	2	N.A.	N.A.	blood
					96	crew 1	2	N.A.	N.A.	
					140	crew 2	2	N.A.	N.A.	
					175	crew 3	2	N.A.	N.A.	
					185	crew 4	2	N.A.	N.A.	
49	Vorob´ev et al. (1986) (87)		USSR	Salyut	149		2	N.A.	N.A.	blood
Group 2: Gravity simulation studies										
50	Bonnefoy et al. (2022) (88)		FRA	HDT-BR	60	–	20	34 ± 8	0.0	blood
51	Jacob et al. (2022) (89)									
52	Brooks et al. (2014) (90)		USA	BR	28	–	31	30-55	0.0	blood
53	Buescher et al. (2024) (91)		GER	HDT-BR	30	–	24	35 ± 8	37.5	blood
54	Chouker et al. (2001) (92)		GER	HDT-BR	120	–	6	31 ± 8	0.0	blood
55	Clement et al. (2022, a) (93)		USA/GER	HDT-BR	60	control	8	33 ± 9	25.0	blood
				HDT-BR	60	IV1	8	33 ± 9	37.5	
				HDT-BR	60	IV2	8	33 ± 9	37.5	
56	Clement et al. (2022, b) (94)		USA/GER	HDT-BR	30	–	12	25-50	50.0	blood
57	Crucian et al. (2009) (95)		USA	HDBR	50	IV-short	4	N.A.	50.0	blood
				HDBR	90	IV-full	6	N.A.		
58	Hoff et al. (2015) (96)		GER	HDT-BR	60	–	24	20-45	0.0	blood
59	Ivanova et al. (2005) (97)		RUS	AOSH (BR)	120	–	6	25-40	0.0	blood
60	Kalandarova (1991) (98)		RUS	AOSH	370	–	N.A.	N.A.	N.A.	blood
61	Lesniak et al. (1998) *(→ ID 28)* (67)		RUS	AOSH	365	–	9	N.A.	N.A.	blood
62	Lesniak et al. (1999) (99)		RUS	AOSH	60-120	–	10	N.A.	N.A.	blood
63	Meshkov et al. (1998) *(→ ID 32)* (72)		RUS	HDT-BR	60-120	–	10	24-40	0.0	blood
64	Novoderzhkina et al. (1996) (100)		RUS	AOSH (BR)	120	–	8	25-37	0.0	blood
65	Schmitt et al. (1996) (101)		FRA/USA	HDT-BR	28	–	6	N.A.	0.0	blood
				BR	119	–	2	N.A.	0.0	
66	Schmitt et al. (2000) (31)		FRA	HDT-BR	42	–	8	N.A.	0.0	blood
67	Shearer et al. (2009) (102)		USA	HDBR	60	control	8	25-40	100.0	blood
					60	IV1	8	25-40	100.0	
					60	IV2	8	25-40	100.0	
68	Sonnenfeld et al. (2007) (103)		USA	HDT-BR	60	–	N.A.	N.A.	N.A.	blood, saliva
69	Trudel et al. (2009) (104)		CAN	HDT-BR	60	–	24	25-40	100.0	blood
70	Uchakin et al. (105)		RUS	HDBR	120	–	6	31 ± 8	0.0	blood
71	Uchakin et al. (2007) (106)		USA	BR	28	–	13	37 ± 9	0.0	blood
72	Volozhin et al. (2001) (107)		RUS	AOSH	60	–	4	N.A.	N.A.	blood, saliva
73	Xu et al. (2013) (108)		CHN	HDBR	45	–	15	27 ± 4	0.0	blood
74	Xu et al. (2016) (109)		CHN	HDBR	45	–	8	27 ± 4	0.0	blood
Group 3: Terrestrial, artificial habitat										
75	Antropova et al. (110)		RUS	HC	30-60	–	26	25-45	0.0	blood
76	Buravkova et al. (2007) *(→ ID 7)* (46)		RUS	PC	240	240	4	27-48	N.A.	blood
				PC	110	110	8		N.A.	
77	Chen et al. (2020) (111)		CHN	CELSS	180	–	4	29-43	25.0	blood
78	Chouker et al. (2002) (112)		GER/RUS	PC	110	110	4	42 ± 6	0.0	blood
				PC	240	240	4	42 ± 6	0.0	
79	Douglas et al. (2022) (113)		USA	HERA	45	–	16	40 ± 9	37.5	blood
80	Hao et al. (2022) (114)		CHN	BLSS	370	–	4	27 ± 2	50.0	saliva
81	Kalandarova et al. (1983) (115)		RUS	HC	31-39	–	11	N.A.	N.A.	blood
82	Konstantinova et al. (1997) (116)		RUS	HC	135	–	3	N.A.	N.A.	blood
83	Larkin et al. (1972) (117)		USA	PC	62	–	8	19-23	0.0	blood
84	Li et al. (2022) (118)		CHN	MARS500	N.A.	–	6	N.A.	N.A.	blood
85	Morukov et al. (2013) (119)		RUS	HC	520	–	6	N.A.	N.A.	blood
86	Nwanaji-Enwerem et al. (2020) (120)		USA	MARS500	520	–	6	N.A.	0.0	blood
87	Rykova et al. (121)		RUS	HC	240	–	4	37-48	N.A.	blood
88	Schmitt et al. (1995) (122)		FRA	PC	60	–	4	26-38	25.0	blood
89	Husson (1996) (123)									
90	Sonnenfeld et al. (1992) (124)		USA/ITA	Cave	131	–	1	27	100.0	blood
91	Strewe et al. (2015) (125)		GER	Space module	105	–	6	33 ± 5	0.0	blood
92	Uchakin et al. (2006) (126)		RUS	PC	120	–	N.A.	N.A.	N.A.	blood
93	Walford et al. (1992) (127)		USA	Biosphere 2	183	–	8	25-67	50.0	blood
94	Xun et al. (2018) (128)		CHN	CELSS	180	–	2	N.A.	N.A.	blood
95	Yi et al. (2014) (129)		GER	MARS500	520	–	6	32 ± 4	0.0	blood
96	Yi et al. (2015) (130)		GER	MARS500	520	–	6	33 ± 6	0.0	blood
97	Yuan et al. (2019) (131)		CHN	CELSS	180	–	4	34 ± 7	N.A.	blood
Group 4: Terrestrial, natural habitat										
98	Allen et al. (1973) (132)		GBR	Antarctica	260	–	14	21-35	0.0	blood
99	Bell et al. (1987) (133)		GBR	Antarctica	≤ 365	–	9	22-37	0.0	blood
100	Bhushan et al. (2019) (134)		IND	Antarctica	90	–	12	32.5 (28-37)	0.0	blood
				Antarctica						
101	Bhushan et al. (2021, a) (135)		IND	Antarctica	48	–	12	22-60	0.0	blood, salvia
				Antarctica	270	–	11		0.0	
102	Bhushan et al. (2021, b) (136)		IND	Antarctica	30	–	12	22-60	0.0	blood, saliva
103	Cameron et al. (1968) (137)		AUS	Antarctica	≤ 365	–	27	N.A.	N.A.	blood
104	Chen et al. (2016) (138)		CHN	Antarctica	352	Station 1	12	40 ± 10	0.0	blood
				Antarctica	391	Station 2	16	34 ± 10	0.0	
105	Diak et al. (2024) (139)		USA	Antarctica	240	–	24	N.A.	N.A.	blood, saliva
106	Evdokimov et al. (1983) (140)		USSR	Antarctica ***^h^***	≤ 365	–	27	N.A.	N.A.	blood
107	Feuerecker et al. (2014) (141)		GER	Antarctica ***^h^***	30	–	9	39 ± 12	0.0	blood
108	Feuerecker et al. (2019) (142)		GER	Antarctica ***^h^***	≤ 270	–	14	36 ± 11	N.A.	blood
109	Feuerecker et al. (2022) (35)		GER	Antarctica ***^h^***	≤ 365	Concordia	39	35 ± 9	17.9	blood
				Antarctica		Neumayer				
110	Flynn et al. (1977) (143)		USA	Antarctica	198	McMurdo	66	N.A.	N.A.	blood
				Antarctica	198	ScottBase	11	N.A.	N.A.	
111	Gleeson et al. (2000) (26)		AUS	Antarctica	≤ 365	Casey	16	23-42	12.5	saliva
				Antarctica		Davis	30	24-55	16.7	
				Antarctica		Mawson	27	26-51	3.7	
112	Hammermeister et al. (1992) (144)		GER	Submarine ***^h^***	N.A.	–	64	20-33	N.A.	blood
113	Holmes et al. (1971) (145)		GBR	Antarctica	≤ 300	–	13	N.A.	0.0	blood
114	Johnsen et al. (146)		NOR	Antarctica	93	–	1	34	0.0	blood
115	Kantorovich (1970) (147)		N.A.	Arctica	≥ 365	–	154	N.A.	N.A.	blood
116	Kovardakov et al. (1976) (148)		N.A.	Swimming	N.A.	–	30	18-30	N.A.	blood
117	Kurbanov et al. (1977) (149)		USSR	Antarctica ***^h^***	≥ 365	–	27	25-47	0.0	blood
118	Lund and Dowdle (1977) (34)		ZAF	Antarctica	275-365	–	21	N.A.	0.0	blood
119	Mehta et al. (2000) (150)		USA	Antarctica	270-300	–	16	26-56	12.5	saliva
				Antarctica						
120	Mishra et al. (2010) (151)		IND	Antarctica	56	–	30	22-60	10.0	blood
121	Mishra et al. (2011) (29)									blood, saliva
122	Mishra et al. (152)									
123	Moraes et al. (2023) (153)		BRA	Antarctica	50	–	7	32 ± 8	28.6	saliva
124	Moraes et al. (2024) (154)									
125	Mrakic-Sposta et al. (2022) (155)		ITA	Antarctica ***^h^***	300	–	13	34 ± 3	23.1	Blood
126	Muchmore et al. (1970) (156)		USA	Antarctica ***^h^***	287	–	5	N.A.	0.0	blood
127	Muchmore and Shurley (1974) (157)		USA	Antarctica ***^h^***	≤ 365	–	18	N.A.	N.A.	blood
128	Muller et al. (1995) (158)		AUS	Antarctica	56	–	29	22-51	6.9	blood
				Antarctica						
129	Nieman et al. (159)		USA	Antarctica	54	–	1	33	0.0	blood
130	Novikov et al. (1991) (160)		N.A.	Antarctica	N.A.	–	205	N.A.	N.A.	blood
131	Roberts-Thomson et al. (1985) (161)		AUS	Antarctica ***^h^***	140	–	12	37 ± 2	0.0	blood
132	Ryabinin (1972) (162)		USSR	Antarctica	≥ 365	–	39	N.A.	N.A.	blood
133	Sakai et al. (2004) (163)		JPN	Antarctica	366	–	39	36 ± 4	0.0	blood
134	Sapov et al. (1981) (164)		USSR	Arctica	≥ 30	–	348	19-35	N.A.	blood
135	Shearer et al. (2001) (165)		AUS	Antarctica	≤ 240	–	11	24-55	0.0	blood
136	Shearer et al. (2002) (166)		AUS	Antarctica	≤ 240	–	21	N.A.	0.0	blood
				Antarctica						
137	Shirai et al. (2003) (27)		JPN	Antarctica	456	–	40	25-50	7.5	blood
				Antarctica ***^h^***						
138	Strewe et al. (2019) (167)		GER	Antarctica	≤ 365	–	16	37 ± 9	0.0	blood
				Antarctica		–	10	31 ± 8	100.0	
139	Tashpulatov et al. (1971) (168)		USSR	Antarctica	N.A.	–	14	N.A.	N.A.	blood
140	Tashpulatov et al. (1976) (163)		USSR	Antarctica	N.A.	–	27	N.A.	N.A.	blood, saliva
				Antarctica ***^h^***						
141	Tingate et al. (1997) (169)		AUS/USA	Antarctica	≤ 365	–	19	N.A.	10.5	blood
142	Yadav et al. (2012) (30)		IND	Antarctica	≥ 365	–	22	25-60	0.0	blood
143	Žákovská (2023) (170)		CZ	Antarctica	60	–	15	25-61	20.0	blood

AUS, Australia; AOSH, Antiorthostatic hypokinesia without further description of the conduction; BLSS, biogenerative life support system e.g. Lunar Palace 1; BR, bed rest (horizontal); BRA, Brazil; CELSS, controlled ecological life support system; CHN, China; CZ, Czech Republic; d, days; f, female; FRA, France; GBR, Great Britain; GER, Germany; h, hypoxic environment; HC, hermetical chamber; HDBR, head down bed rest; HDT, head down tilt; HERA, Human Exploration Research Analog; IND, India; ISS, International Space Station; ITA, Italia; IV, intervention; JPN, Japan; NOR, Norway; N.A., not applicable; PC, pressurised chamber; RUS, Russia; USA, United States of America; USSR, Union of Soviet Socialist Republics; y, year; ZAF, Republic of South Africa.

The 140 included publications date from 1968 to 2024 and data were mainly published by Asian researchers (42%), followed by American (26%) and European research teams (19%). We also analyzed publications from Australian (4%) and African (1%) researchers, as well as engaging insights from international collaborations (5%). Additionally, 3% of the publications did not provide sufficient information to determine the region of origin.

Several publications have analyzed data from the same cohorts, applying different research questions and methodologies to explore various aspects of immune function. In the Twin Study conducted by the National Aeronautics and Space Administration (NASA), Garrett-Bakelman et al. (52), Bezdan et al. (53), and Gertz et al. (54) focused specifically on changes in T cell counts, immunoglobulin counts, and cytokine counts, respectively. Similarly, Kimzey et al. examined immunoglobulins, complement factors, and lysozyme in the Skylab 3 cohort (57), later expanding to neutrophil and lymphocyte counts (58). Vorobyov et al. (85) and Vorob’ev (86) et al. reported on the same Soyuz mission, with the former focusing on in-flight changes in T lymphocytes and Natural Killer (NK)-cells, while the latter examined pre- and post-mission immune changes. Kuzichkin et al. (65) reported on monocytes and granulocytes from a mission on the ISS and later used a method of higher quality (66), enhancing the reliability of their previous findings. In gravity simulation studies, Bonnefoy et al. (88) and Jacob et al. (89) analyzed lymphocyte and neutrophil counts across all time points, differing mainly in the timing of baseline and post-mission measurements. Bonnefoy et al. also included immunoglobulin data, providing additional insights (88). Schmitt et al. (122) and Husson (123) compared immune cell subsets, including T and B lymphocytes and NK cells, in a pressurized chamber. Mishra et al. conducted multiple analyses on 30 participants from an Antarctic research station, focusing on cytokines, chemokines (151), and immunoglobulins measured in both serum (29) and saliva (152). Finally, Moraes et al. (153) published cytokine data from research in Antarctica (153) and, a year later, supplemented their report (154) with additional findings, including data on the acute-phase protein Serum Amyloid A (SAA). Studies involving the same participants were analyzed as a single cohort to avoid double-counting errors and ensure consistency.

In contrast, three studies spanned multiple groups due to investigations in both space and bed rest (67, 72) or pressurized chambers (67), which is why some studies are listed in several groups. We report on these studies separately, which is why we refer to 143 studies in the following.

We thus examined a total of 2,700 healthy participants (> 90% male) with a median number of subjects/mission of 10 (range: 1-348), covering the ages between 18 and 67 years. Missions spanned from 28 to 520 days. However, the precise duration in Antarctic studies is often indeterminate, suggesting that some missions may have extended beyond 520 days. The isolation period itself was examined in 82% of the studies. Fewer studies reported on the recovery period (57%) or on pre- and post-isolation comparisons (69%). Most studies were conducted in space (Group 1, n=49 (40–87, 171), mainly on the ISS (55%). Space analogue studies were mostly done in terrestrial natural environments (Group 4, n=46 (26–30, 34, 35, 132–170)), primarily in Antarctic research stations (91.3%), with 26.1% taking place in hypoxic conditions. Less than 10% occurred outside Antarctica. Additionally, n=23 (46, 110–131) studies were in terrestrial artificial environments (Group 3) and n=25 (31, 67, 72, 88–109) in gravity simulations (Group 2).

### 3.2 Individual analysis of the habitats

#### 3.2.1 Space missions

**Figure 2:**
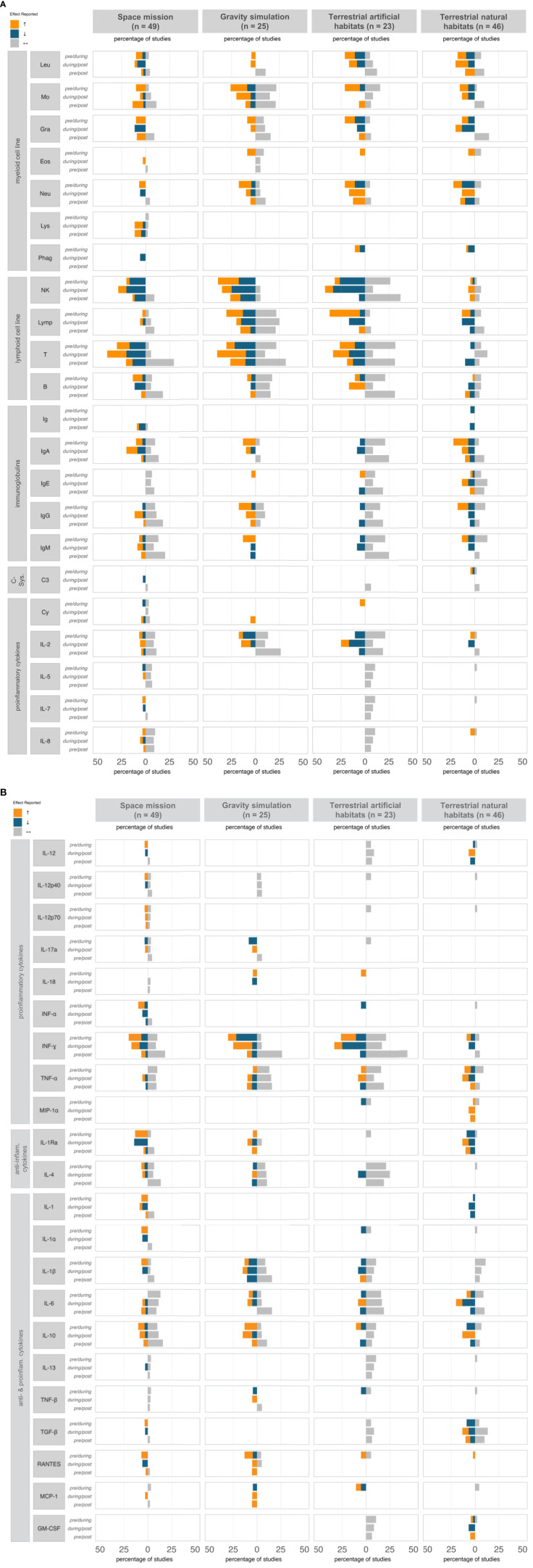
Immune response profile (% reports) at different time points, classified by ICE condition. The diagram illustrates the percentage of studies reporting effects on various immunological parameters—categorized as increase (↑, orange), decrease (↓, blue), or stability (↔, grey)—across three time points: pre- vs. during isolation, during vs. post isolation, and pre- vs. post isolation. For clarity, only immune parameters with a ≥5% reporting frequency were included; unchanged parameters are not shown. anti, anti-inflammatory; B, B-cells; C, Complement component; Cy, Cytokines; Eos, Eosinophils; GM-CSF, Granulocyte Macrophage Colony-Stimulating Factor; Gra, Granulocytes; Ig, Immunoglobulin; IL, Interleukin; IFN, Interferon; Leu, Leukocytes; Lymp, Lymphocytes; Lys, Lysozyme; MCP, Monocyte Chemoattractant Protein; MIP, Macrophage Inflammatory Protein; Mo, Monocytes; NK, Natural Killer cells; Neu, Neutrophils; Phag, Phagocytes; RANTES, Regulated on Activation, Normal T cell Expressed and Secreted; T, T-cells; TGF, Transforming Growth Factor; TNF, Tumor Necrosis Factor.

In space missions, considerable heterogeneity was observed in both the selection of immunological parameters assessed and the patterns of immune markers reported. Nevertheless, some tendencies regarding immune response might be suggested (**Figure 2**, **Supplementary Tables S1**-**S4**). Specifically, the myeloid lineage was mostly reported to increase during spaceflight, decline post-flight, and remain stable in the pre-/post comparisons, while immunoglobulin levels remained stable throughout all time points. Irrespective of their pro- or anti-inflammatory classification, cytokines increased or remained stable during the mission and decreased or remained stable post-mission, while overall pre- and post-mission levels showed no substantial changes. Most changes were observed in the lymphoid lineage, with increases and decreases occurring to a similar extent during the mission, a tendency toward more decreases post-mission, and overall pre- to post-mission stability. Over 70% of studies reported a post-mission return to baseline.

Specific trends in immune parameters were observed, with increases in granulocytes, particularly neutrophils, B cells, cytokines (e.g., IL-1, IL-3, IL-7, IL-12, IL-15, Interferon (IFN)-α, Tumor Necrosis Factor (TNF)-α, IL-1Rα, IL-1α, IL-1β, Transforming Growth Factor (TGF)-β, CC-Chemokine Ligand 5 (RANTES)), and Insulin-like Growth Factor (IGF)-1 during missions. While most of these parameters tended to return to baseline levels and remained stable in pre-/post-mission comparisons, granulocytes and RANTES exhibited a more variable response and did not fully stabilize post-mission. Notably, NK cells and IL-3 decreased throughout the isolation period and continued to decline post-isolation, indicating potentially sustained immune effects. In contrast, IgD, IgE, IgG, IgM, IL-2, IL-5, IL-8, IL-17, TNF-α, Macrophage Inflammatory Protein (MIP)-1β, IL-4, IL-6, IL-10, and TNF-β were reported to remain unchanged across all time points, suggesting a potential subset of immune markers that were unaffected by spaceflight conditions.

#### 3.2.2 Gravity simulation

Although fewer gravity simulation studies were conducted compared to space missions, certain immunological measures were investigated more frequently. However, this did not lead to clearer trends, as the results remained highly heterogeneous, limiting the ability to draw expressive conclusions (**Figure 2**, **Supplementary Tables S1**-**S4**). Most studies focused on lymphoid cell lineage outcomes; however, reporting frequencies for increases, decreases, and stability were relatively balanced, indicating considerable variability in lymphoid cell responses. A similar heterogeneity was reported for cytokines, regardless of their pro- or anti-inflammatory classification; nevertheless, both lymphoid cell lineage and cytokines exhibited stability in pre-/post comparisons. Overall, about 70% of all studies reported pre-/post-stability in immunological parameters, with monocytes, T lymphocytes, IL-2, and IFN-γ remaining stable in a significant portion of studies.

Compared to space missions and other space analogues, gravity simulation studies exhibited fewer consistent patterns across time points on parameter level. Specifically, NK cells, IL-18, and IgM levels increased during the study, followed by a post-simulation decline, while IL-17α, Monocyte Chemoattractant Protein (MCP)-1, IFN-γ, and TNF-β consistently decreased during gravity simulation. However, neither trend was reported as fully stabilized, as most studies reported only partial recovery post-simulation. Lymphocytes, in particular B cells, TNF-α, and IL-4 remained unchanged across all time points, indicating unaffectedness under simulated microgravity. Comparable clear patterns were not observed for other immunological measures.

#### 3.2.3 Terrestrial, artificial isolation

Terrestrial space analogue studies, such as MARS-500 or comparable programs, measured fewer immunological parameters than other groups but exhibited the largest magnitude of changes, likely due to longer mission durations. Nearly half of the studies reported consistency in NK cells and IFN-γ between pre- and post-mission, indicating a clear trend for resilience (**Figure 2**, **Supplementary Tables S1**-**S4**). While effects (increase or decrease) were predominant during and after missions, artificial chamber simulations showed overall less distinct differentiation between immunological parameters showing change and those remaining stable compared to other groups. Specifically, 54% of studies on terrestrial, artificial isolations reported stable immune parameters during the mission, and 46% observed no significant changes post-mission. Considerable increases during mission were only noted in myeloid cell counts, while lymphocytes showed mixed effects. However, the immunological parameters remained unchanged in the pre-/post-comparison, with 80% of studies reporting a return to baseline levels after the mission.

In terrestrial artificial isolations, a single clear parameter pattern effect emerged. Lymphocyte numbers increased during isolation, followed by post-mission decreases, but a consistent return to baseline levels was not explicitly confirmed. However, IgE, IgG, IL-5, IL-7, IL-8, IL-12, IL-6, IL-13, Granulocyte Macrophage Colony-Stimulating Factor (GM-CSF), and TGF-β were reported to remain unchanged across all time points, suggesting potential unaffectedness of these kind of space analogues, with some overlaps to stability findings in space missions.

#### 3.2.4 Terrestrial, natural isolation

In terrestrial natural habitats, there were fewer reported immunological parameters and smaller amplitudes in the reported immunological effects (**Figure 2**, **Supplementary Tables S1**-**S4**). Research predominantly focused on the myeloid cell lineage and immunoglobulins, and uniquely examined the role of the complement system in this habitat. Reports indicated increased complement component concentrations during isolation but provided limited evidence of a return to baseline, suggesting possible sustained effects; however, too few studies have investigated complement-related factors to support an evidence-based conclusion. In the pre-/during-comparison, both increasing and decreasing incidences of the analyzed immunological parameters were reported, with the ratio of these incidences remaining the same post-mission, though only lymphocytes in general, IL-2 and IL-10 displayed clear inverse patterns (lymphocytes and IL-2 increased during isolation and decreased post-isolation, while IL-10 showed the opposite). IL-1 levels showed a consistent decline that persisted post-isolation, while other immunological measures demonstrated no clear trends, resulting in an overall heterogeneous profile. Despite this, the pre-/post-comparison revealed that the majority of the reported immunological measures (52%) remained unchanged, suggesting a lack of sustained effects.

**Figure 3:**
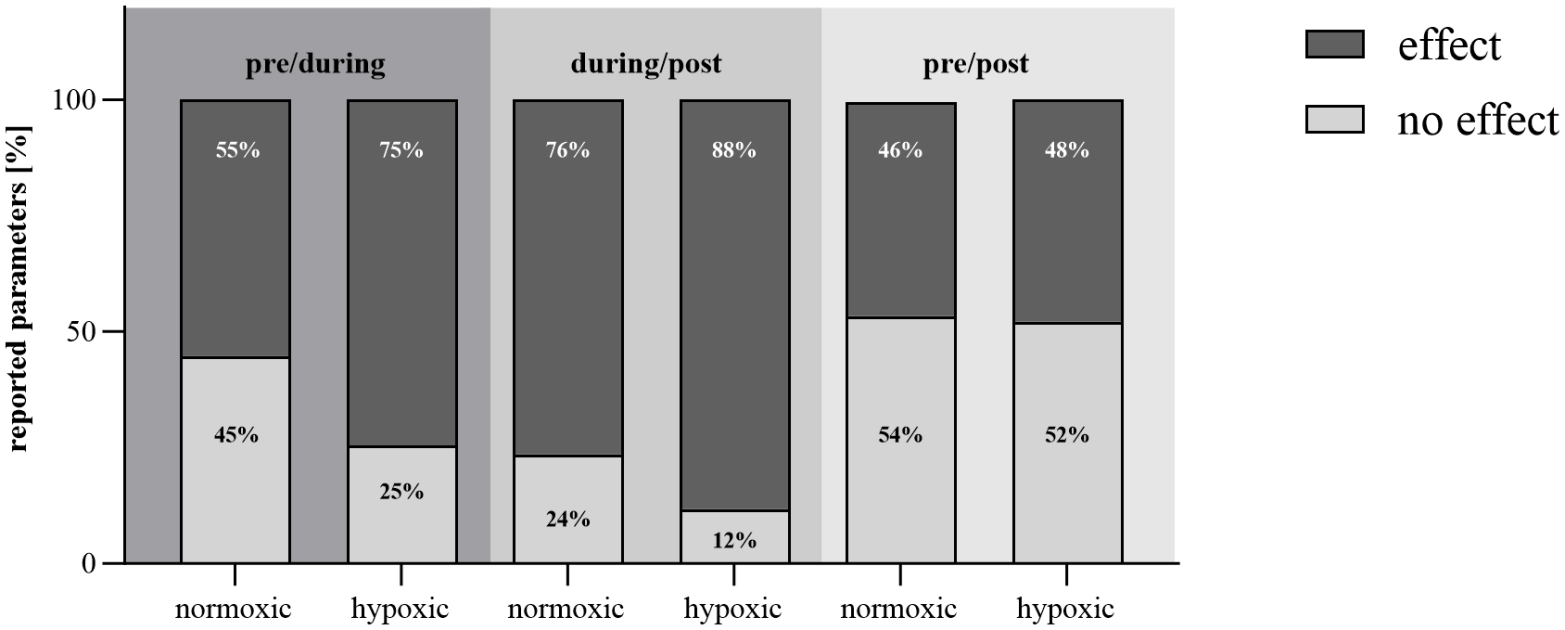
Effects of normoxic and hypoxic conditions on immunological parameters. Bar chart illustrating the reported effects in immunological parameters (% of all reported effects) within terrestrial habitats under normoxic and hypoxic conditions, categorized by their occurrence pre/during, during/post, and in pre/post comparison of mission periods. The chart quantifies the percentage of studies noting an effect (dark grey), and no change (light grey) in immunological responses.

In addition to isolation and stress factors, intermittent hypoxic conditions were also present, likely influencing immune responses. To distinguish the effects of hypoxia from isolation on immune dysregulation, a subgroup analysis was conducted (**Figure 3**). Under both normoxic and hypoxic conditions, isolation was associated with fluctuations in immunological measures, with a slightly higher incidence of changes observed under hypoxia. Post-isolation, reports of immune alterations increased under both conditions compared to during the isolation phase, with hypoxia showing the highest prevalence of effects (increase or decrease); however, no consistent trend in immune response emerged. In the pre-/post-comparison, stability increased compared to the effects seen during isolation, suggesting a trend toward equilibrium with no significant changes in immune responses regardless of oxygen levels. Overall, these findings indicate that although hypoxic conditions marginally intensified immune fluctuations, both environments supported a return to baseline, reflecting resilience in immune recovery.

### 3.3 Combined analysis of the habitats

The percentage changes and stabilities for each immunological measure across all habitats and time points is summarized in **Figure 2**, with further information available in **Supplementary Table S1**. The detailed results of each study are described in **Supplementary Tables S2**-**Supplementary Tables 4**. Across all groups, the primary immune markers evaluated in terms of production included total T cells, NK cells, and IFN-γ. Overall, most studies reported that immune markers remained stable in the pre-/post-comparison.

Across all included studies, and irrespective of ICE conditions and assessment time points, most changes or consistencies were reported for the lymphoid cell lineage, with an average reporting frequency of 8.9% per immunological parameter. During the isolation phase, no definitive trends were observed, with immunological parameters showing low differences in reporting frequencies for increases (32.8%), decreases (24.8%), and stability (42.4%). Conversely, the pre-/post-comparison revealed a predominant pattern of stability, with 68.2% of immunological parameters reported as stable. Especially, studies reported clearly on T cell stability in the pre-/post-comparison (56.8% of all studies reporting on T cells), with tendencies to rise in the recovery phase (45.5% of all studies reporting on T cells). In contrast, NK cells exhibited an opposing pattern during the recovery phase, with the majority of studies indicating a downward trend (61.5% of all studies reporting on NK cells). B-cell levels, however, were rarely reported and generally remained stable, irrespective of time points.

Notably, myeloid cell factors and immunoglobulins were frequently examined, with average frequencies of 3.7% and 3.9%, per immunological parameter respectively, across all included studies. The most commonly reported myeloid parameters included leukocytes, monocytes, granulocytes, especially neutrophils, while for immunoglobulins, the focus was on IgA, IgG, and IgM. Overall, no clear patterns of significant change were observed during the isolation or recovery phases, as findings varied, with some studies showing increases and others reporting decreases or stability. However, pre-/post comparisons predominantly indicated stability, with 58.0% of studies reporting on myeloid cell lineage pre-/post comparisons finding stability, and 74.3% of studies analyzing immunoglobulins in pre-/post comparisons also reporting no change, even during isolation.

Moreover, several studies reported on various proinflammatory cytokines, with an average reporting frequency of 1.6% per immunological parameter. The most frequently assessed were IL-2, TNF-α, and IFN-γ. Specifically, studies reported a similar ratio for IFN-γ with increases, decreases, and stability, while IL-2 and TNF-α remained largely stable throughout and after the isolation period. Overall, significant changes during the isolation or recovery phases were not evident. When changes were observed, they were often contradictory within the same immunological parameter, with some studies showing increases and others reporting decreases or stability. This aligns with the overall stability reported in pre-/post comparisons, with 82.4% of studies analyzing IL-2 in pre-/post comparisons reporting stability, 68.8% for TNF-α, and 77.8% for IFN-γ. Similar adaptation patterns were observed for anti-inflammatory cytokines, further supporting the consistency of these findings.

The effects involving the complement system components were less commonly reported (average reporting frequency per immunological parameter: 0.3%). Due to the limited number of studies addressing complement system factors, further analysis or discussion of its role is not further pursued.

**Figure 4:**
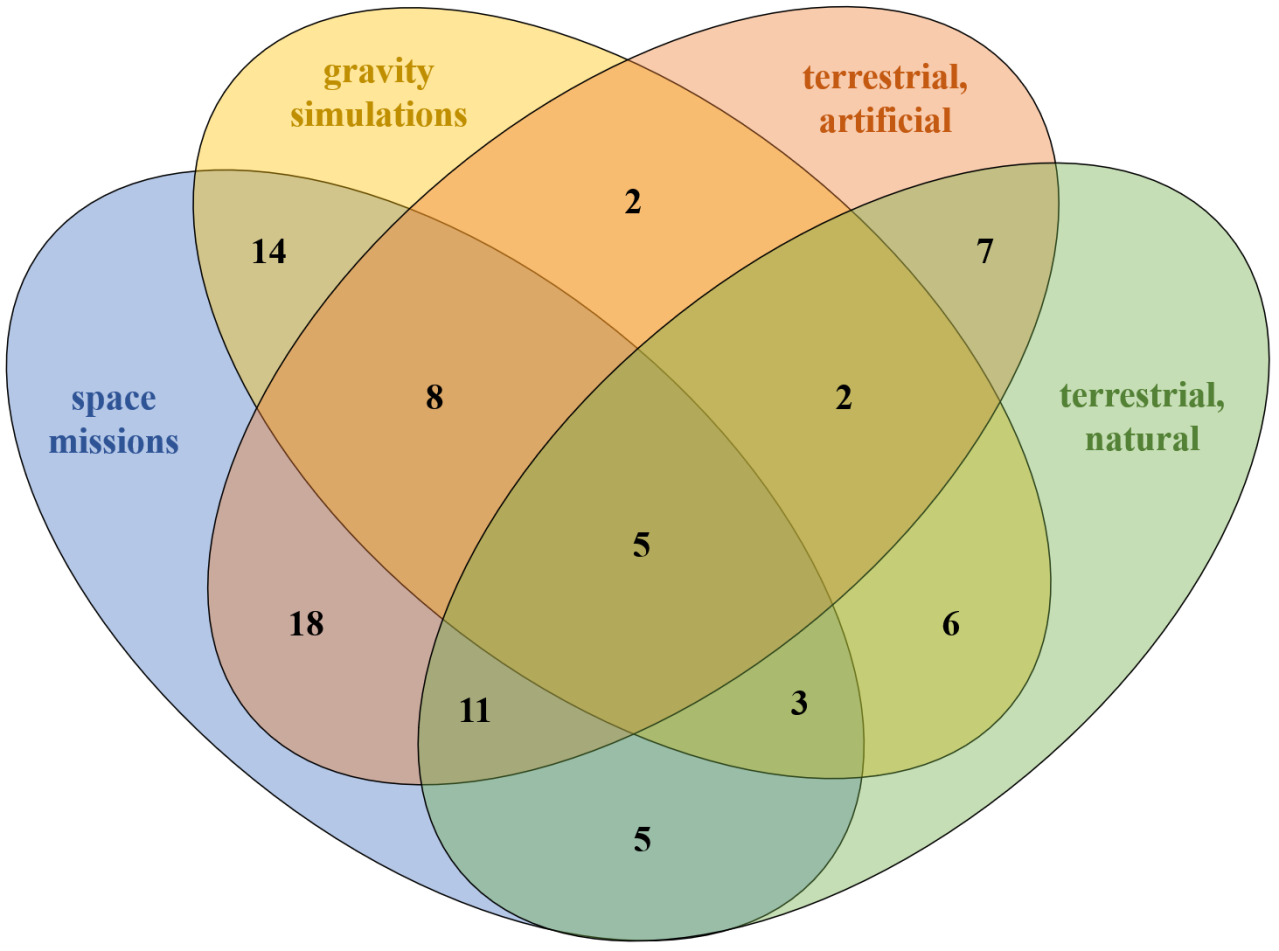
Similarities in absolute values between the ICE conditions. Similarity means that either all studies indicated the same direction (increase, decrease or unchanged) or the majority of studies indicated the same direction.

Owing to the great heterogeneity resulting from the extremely different study designs, numbers of participants, mission durations and reporting frequency of immunological measures, general outcomes across the habitats were rare and unspecific. There were the same effects for five markers (**Figure 4**), which was shared by all groups, namely the stability of IL-2, IL-4, IL-6, TNF-α and IFN-γ in the pre-/post-comparison. Although there was little overlap in overall immune response patterns across time points, individual effects showed substantial similarity between space missions and terrestrial artificial isolation studies, with 42 shared effects—most notably the decline in NK cell levels during isolation and their sustained suppression post-isolation. These similarities indicate potential common immune responses between space and tightly controlled artificial environments, where limited antigen exposure, among other possible stressors, may contribute to the observed effects. Distinct parallels were also observed between space missions and gravity simulation studies, though these were similarly limited to stable parameters independent of time point. Overall, there was a remarkably low level of overlap in immunological measures between gravity simulation studies and terrestrial isolation missions. Notably, immune effects were comparable between gravity simulation studies and terrestrial natural conditions, and 17 with artificial terrestrial conditions (**Figure 4**). However, similarities between two groups were largely confined to immunological measures that remained stable across all time points.

### 3.4 Clinical outcomes, allergies and medication reports

**Figure 5:**
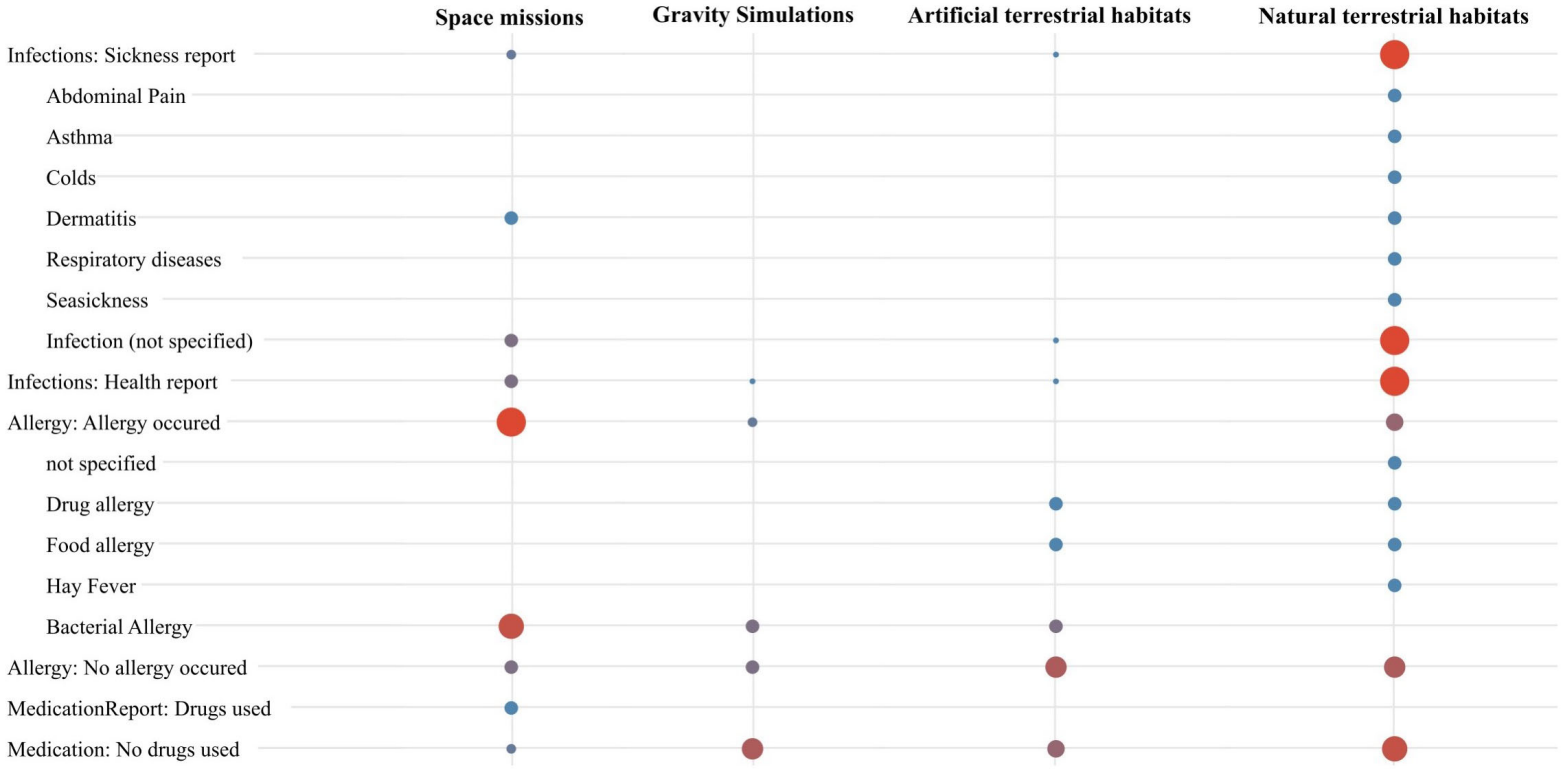
Summary of reported health outcomes across different habitats in all studies. This visualization presents a comparative analysis of health outcomes, illustrating the varying frequencies of reported infections, allergic reactions, and medication use across diverse environmental conditions. Larger red points indicate higher reporting frequencies, while smaller blue points represent lower frequencies. The calculation of symbol size refers to the row. Studies that made no mention of health-related outcomes were excluded from this representation.

Only 40 of 140 studies documented clinical outcomes related to infections (**Figure 5**). In most studies conducted in natural terrestrial habitats, respiratory diseases were prevalent among isolated individuals, manifesting as acute chest infections, persistent sinusitis, and nasopharyngitis. Mild respiratory symptoms, including cough and sore throat, were commonly observed post-departure, frequently lasting one to two weeks. Seasonal rhinitis and recurring colds, particularly during the re-adaptation phase, were also noted, with the latter resembling epidemic outbreaks following contact with new personnel, likely due to exposure to novel antigens. General infections were identified, encompassing bacterial infections, gingivitis, and otitis media. Additionally, one case of asthma (169), one case of herpes virus (139) and instances of seasickness (29), particularly at the onset of isolation, were documented. Abdominal pain and dyspeptic symptoms were occasionally reported, often following re-exposure to external personnel, likely due to introduced antigenic diversity. In a space study, cosmonauts who were non-carriers of *S. aureus* prior to flight exhibited colonization of the nasal cavity, mouth, and pharynx after 6 to 18 days in space (75), accompanied by reports of both symptomatic and asymptomatic dermatitis (69). A recent case report described an astronaut who spent six months on the ISS and, in addition to spaceflight-associated neuro-ocular syndrome, also experienced symptoms such as rashes and headaches, which were later diagnosed as a Zoster infection (70). Similarly, in a pressurized chamber with poor air quality conditions (elevated ammonia and CO_2_), viral infections developed in 8 of 12 isolated subjects, with two presenting symptoms such as fatigue, lethargy, sore throat, and skin rashes (110). However, in a recent study conducted at NASA’s Human Exploration Research Analogue, 16 participants were tested for herpes viruses, including EBV, herpes simplex virus type 1 (HSV-1), and VZV (113). Although EBV and HSV-1 shedding was detected, levels remained below clinical concern and were lower than changes typically associated with spaceflight (50). While 17 studies confirmed that medical examinations identified no diseases, 100 studies did not assess or report the presence of potential infections.

Immune sensitizations and clinically manifested allergies were primarily reported in the context of space missions and natural habitats. Overall, 6 of 49 studies conducted in space identified findings related to allergies, with some cosmonauts exhibiting sensitization to bacterial allergens, particularly *Streptococcus* and *Staphylococcus* (59–61, 67, 86, 87). In 3 of 46 Antarctic studies (34, 35, 161), participants demonstrated type I hypersensitivity during the stay or after return, along with food and penicillin allergies, as well as hay fever that resolved post-return. These participants also showed sensitivities to grass pollens, cat fur, and increased reactivity to purified protein derivative. In another study, participants self-reported perceivable allergy symptoms; however, these allergies had already been diagnosed before the mission; no new-onset allergies occurred during the mission (139). Additionally, two studies conducted in closed chambers reported sensitization to tuberculin without clinical symptoms (116), as well as to various plant and food allergens, including plantain pollen, rye pollen, shrimp, and rye flour (121). One study documented allergies in the context of bed rest (67), another one indicated that allergies induced by bed rest conditions could be ruled out, as the limited increase in IgE levels was not accompanied by a corresponding rise in eosinophil counts (88). Notably, 127 studies did not report any allergy-specific outcomes.

Medications were not reported in more than 90% of the trials. In the few studies where medication use was reported, there were no medications mentioned that could significantly affect immunological parameters. Only the case report mentioned the use of an antiviral medication and ibuprofen (70).

### 3.5 Risk of bias

**Figure 6:**
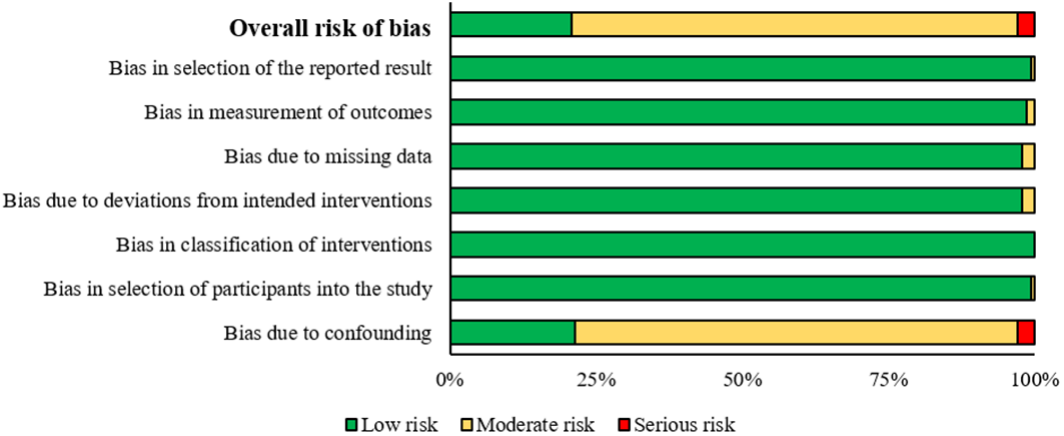
Risk of bias analysis, summary plot.

The risk of bias across the studies included in the review is presented in **Figure 6**, and more detailed in the **Supplementarys Figure S1**. Among the 140 non-RCTs, none was considered to have critical risk of bias and 4 were at serious risk of bias due to confounding bias. Only 21% of the studies included were considered to have a low risk of bias. A moderate risk of bias was commonly attributed to bias due to confounding, which is why the highest number of studies provides a moderate risk (76%). Notably, the risk of bias due to confounding appears to be the most concerning, with a moderate risk in the majority of studies and a serious risk evident in a smaller but significant proportion of studies (**Figure 6**). However, no study was excluded for a risk of bias.

## 4 Discussion

This review analyzed 140 articles examining how the immune system changes during and after exposure to antigen-limited ICE environments. Overall, immune dysregulation was widely reported in these settings, both during and after isolation; however, considerable heterogeneity persisted across reporting frequencies of immunological parameters, study outcomes, and study durations, even with habitat-specific grouping. A substantial body of evidence already links immune dysregulation to spaceflight (24), and our systematic review not only confirms but further extends this, showing that immune alterations occur across all ICE environments, not only in spaceflight. Given the generally high comparability between space missions and analogues (12, 20), we anticipated observing consistent patterns in immunological measure changes that could allow for extrapolation from analogue environments to actual spaceflight. Overall, lymphoid cell responses showed a tendency toward reduced or increased lymphocyte numbers during isolation, followed by a return to baseline levels post-isolation, resulting in transient and reversible changes. *In vitro* studies from Cogoli and colleagues have revealed, that disturbances of the immune system in gravity-dependent as the microgravity environment from spaceflight causing a consensual decrease in lymphocyte proliferation rates (172). Furthermore, a decrease in T cell numbers (173) may coincide with various factors, including psychological stress or radiation exposure (174). In support with these findings, our results show that in gravity-independent environments (simulation units, Antarctica), T cell levels remained rather stable than showing effects (increasing/decreasing). Considerable stability was observed for immunoglobulin levels in the pre-/post-comparison, especially in space missions (group 1), artificial terrestrial habitats (group 3), and natural terrestrial isolations (group 4). There were few reports of immunoglobulin changes in gravity simulation studies (group 2), and little evidence of a stability during and after the mission or in pre-/post-comparison. Contrary, Crucian and colleagues found that immunoglobulins remained stable during bed rest studies (175). However, while prolonged bed rest serves as a useful analogue for bone loss and muscle deterioration due to disuse (24, 176), it appears rather ineffective in simulating the primary suspected causes of spaceflight-associated immune dysregulation, such as physiological stress, antigen limitation, radiation, and disrupted circadian rhythms.

This review explores the dynamics of immunological measures, focusing on both changes and stability. Since continuous and diverse antigenic stimulation strengthens immune function (8, 9), we anticipated that habitats with greater antigen limitation (space missions, Antarctica) would exhibit more pronounced and severe effects on immune dysregulation. Compared to space missions and studies conducted in natural habitats, bed rest studies and artificial simulations report a higher frequency of stable parameter levels, although space mission and Antarctica studies have a rather small alignment in specific immunological parameter pattern alterations. This pattern suggests that the primary drivers of ICE-associated immune dysregulation are more likely linked to environmental influences like antigen limitation, physiological stress, environmental hazards, and disrupted circadian rhythms, rather than bone loss or muscle deterioration due to disuse as from bed rest studies.

Some Antarctic winter-over studies conducted at extreme altitudes may have more far-reaching effects on the immune system (33, 177), potentially explaining discrepancies observed between spaceflight and Antarctic research (24). However, in this analysis, the hypoxia-related effects identified were minimal, suggesting that hypoxia has a lesser impact on immune dysregulation. Furthermore, our findings reveal an alignment between space missions and artificial terrestrial isolations, such as MARS500, where antigen-limited environments and some factors like physiological stress or disrupted circadian rhythms are prevalent. Potentially, the deep and prolonged reduction of microbial exposure, along with physiological stress, could serve as key determinants of immune alterations (178, 179), although other environmental hazards should not be excluded to be key determinants. Alternatively, extreme stressors might intensify immunological changes. For example, *in vitro* studies have demonstrated that gravitational forces can modulate the immune system through mechanical signaling mediated by mechanosensitive factors present on immune cell membranes (174, 180). Additionally, microgravity may impact microbes to be more likely to cause diseases. Immune impairments have also been noted under extreme conditions such as hyperbaric environments, blast shock, radiation exposure, magnetic fields (20), and extreme heat (181), which could increase the risk of infections and reduce the ability to combat pathogens. The complexity of immune responses necessitates further research to disentangle the specific environmental factors influencing these variations (12, 20, 21).

The return of most immunological parameters to baseline levels after missions suggests the reversibility of immune dysregulation in response to changing environmental conditions. Consistently analyzed immune markers, such as T cells, NK cells, and IFN-γ, remained largely constant, reinforcing the potential for reversible immune dysregulation. The high reporting frequency of these markers across studies lends confidence to their significance compared to other immunological parameters. Their resilient quality and capacity for self-regulation and recovery over time have also been established in previous studies (20, 182).

An evidence report from NASA’s Human Research Program (24) has highlighted immune dysregulation as a significant risk to astronaut health and performance during prolonged spaceflight. If immune dysregulation persists in space environments, potential clinical risks may include hypersensitivities, allergies, autoimmunity, increased infection rates, and malignancies associated with impaired tumor surveillance (24). Our analysis supports this concern, particularly in light of the observed decrease in NK cells during space missions. Given their critical role in controlling viral infections and providing early immune defense, this reduction may contribute to the higher infection rates reported in astronauts (24). Furthermore, despite the fact that a large number of studies in this literature review did not address infection reports, it remains indisputable that deep space exploration and its respective analogues pose a significant and serious risk to human health during both short- and long-term missions (24, 70, 183). A NASA short-term study established a link between immune dysregulation and viral reactivation, suggesting that while latent viruses reactivation is generally not a clinical concern on Earth, persistent reactivation during long-duration space missions could pose health risks (183). Similarly, a case report from a long-term ISS mission described an astronaut developing clinical zoster, indicating VZV reactivation during spaceflight, with viral markers returning to baseline within 30 days post-landing (70). Supporting this, we found diagnosis of viral reactivation only in natural ICE environments (e.g., space (70) and Antarctica (139, 160, 169)). In contrast, viral reactivation observed in recent artificial isolation studies was considered clinically insignificant, with viral shedding levels significantly lower than those observed in space (50, 113). This suggests that the stressors in artificial settings may not fully replicate those responsible for reactivation in natural ICE environments. However, considering the rather limited data from long-term missions in different ICE environments on viral reactivation, infections, and other health impairments, as well as their potential long-term consequences, further investigation is required.

Despite these challenges, the literature specifically addressing infections and allergies indicates that both are likely to occur, with infections related to immune dysregulation appearing to be reversible. Reports of immune sensitizations/allergies are more prevalent in space missions and Antarctic studies, while they are largely absent in bed rest studies. This suggests that the isolation and unique conditions of Antarctica and space may significantly contribute to clinical symptoms, indicating that Antarctic winter-over studies could provide a more appropriate terrestrial analogue for long-duration spaceflight than other examined groups. The reported allergens also differ; space missions highlight sensitization to bacteria such as *Staphylococcus* and *Streptococcus*, whereas Antarctic studies focus on more common allergens like pollen, pet dander, and penicillin. However, no significant connections between these reports were established, leaving knowledge gaps regarding the clinical relevance of immune alterations in ICE environments.

### 4.1 Strengths and weaknesses of the systematic review

Overall, this systematic review has several strengths and limitations. A clear strength is the methodological approach taken according to PRISMA (36) and Cochrane criteria. In order to obtain as broad as possible a knowledge of the current data situation, a very specific search term was used which was superior to broader search terms; however, 140 articles could be included in the analysis. Despite clear eligibility criteria, the heterogeneity of the studies was high at study design and study outcomes. To counter this problem, subgroup analyses considering environment and immunological measure clusters were performed which reduced heterogeneity to some degree. While this review specifically focuses on immunological responses, broader phenomena such as accelerated aging or other multidisciplinary outcomes fall outside its scope and would require a more comprehensive approach across multiple domains. Despite differences in habitats, study designs and frameworks for well-conducted studies, all studies were rather highly controlled, which is also reflected in the risk of bias. Here, the ROBINS-I tool for assessing risk of bias in non-randomized studies of interventions, recommended by the Cochrane Handbook, was used.

One of the main issues in the studies reviewed was the extremely low sample sizes, making it challenging to conduct quantitative analyses at the individual study level. To address these limitations, future studies should aim to increase their sample sizes to enhance statistical power. Comparability was also compromised by the analysis of different immune measures with varying levels of detail in subclassification (e.g., leukocytes, lymphocytes, immunoglobulins). By comparing percentage changes or stability levels of the immunological parameters, an attempt was made to present these general tendencies. Besides, the included studies focused on mid to long-term immune system adaptations, which did not allow for insights of short-term regulations. To better understand the effects of long-term living in ICE environments on the human immune system, researchers should consider internal and external (extreme) factors. This will enable a much clearer differentiation between the effects of isolation and those stemming from other variables. Finally, this systematic review is the first of its kind, providing new insights into the effects of isolation on the human immune system.

## 5 Conclusion

This review highlights immune dysregulation across ICE environments, with marked changes in settings characterized by limited antigen diversity, such as space missions and Antarctic habitats. These environments likely drive immune alterations through restricted antigenic exposure, physiological stressors, and disrupted circadian rhythms. Reduced antigen stimulation appears to foster immune “amnesia,” potentially compromising immune memory and reducing resilience to pathogens upon re-exposure. Observed lymphocyte reductions and post-isolation recovery suggest that some immune alterations may be reversible. However, persistent immune dysregulation observed in space missions could heighten susceptibility to infections and hypersensitivities. Given the antigen-limited conditions of Antarctic winter-over or terrestrial, artificial studies, they may serve as more accurate terrestrial models for assessing immune risks associated with extended spaceflight than bed rest analogues. Future research should focus on identifying specific immune vulnerabilities in these settings and developing targeted countermeasures to protect immune health during long-duration missions, ultimately mitigating health risks for both space travelers and individuals in similar terrestrial environments and other vulnerable populations on Earth such as the elderly or immunocompromised individuals, helping to mitigate immune risks in confined settings.

## Data Availability

The original contributions presented in the study are included in the article/**Supplementary Material.** Further inquiries can be directed to the corresponding author.
