# Supplementary material for: Effects of isolated, confined and extreme environments on parameters of the immune system - a systematic review
Source: Front Immunol. 2025 Mar 25;16:1532103. doi: 10.3389/fimmu.2025.1532103 (PMC11975566; doi:10.3389/fimmu.2025.1532103)
Supplement: Supplementary file 1 [file DataSheet1.pdf]

Supplementary Material

Supplementary Text S1: Search Strategy.

Database	Search term
**PubMed** (22.11.2022; 28.01.2025)	((("Antarctic Regions"[MeSH Terms] OR "Antarct*"[Title/Abstract] OR "Arctic Regions"[MeSH Terms] OR "Arctic*"[Title/Abstract] OR "Sub-Antarctic*"[Title/Abstract] OR "Space Simulation"[MeSH Terms] OR "Ships"[MeSH Terms] OR "Moon"[MeSH Terms] OR "Mars"[MeSH Terms] OR "Spacecraft"[MeSH Terms] OR "Extraterrestrial Environment"[MeSH Terms] OR "Biosphere 2"[Title/Abstract] OR "Envihab"[Title/Abstract] OR "Mars-500"[Title/Abstract] OR "Mars-105"[Title/Abstract] OR "SFINCSS-99"[Title/Abstract] OR "Concordia Station"[Title/Abstract] OR "Zhongshan Station"[Title/Abstract] OR "Neumayer"[Title/Abstract] OR "Maitri Station"[Title/Abstract] OR "Great Wall Station"[Title/Abstract] OR "Davis Station"[Title/Abstract] OR "McMurdo Station"[Title/Abstract] OR "Palmer Station"[Title/Abstract] OR "South Pole"[Title/Abstract] OR "Arctowski"[Title/Abstract] OR "Vostok Station"[Title/Abstract] OR "Syowa Station"[Title/Abstract] OR "HI-SEAS"[Title/Abstract] OR "polar institute*"[Title/Abstract] OR "AGBRESA"[Title/Abstract] OR "SIRIUS"[Title/Abstract] OR "Space Station"[Title/Abstract] OR "Apollo"[Title/Abstract] OR "Lunar Palace"[Title/Abstract] OR "submarine*"[Title/Abstract] OR "land based simulator*"[Title/Abstract] OR "Space condition*"[Title/Abstract] OR "long duration space exploration mission*"[Title/Abstract] OR "polar expedition*"[Title/Abstract] OR "Space Flight"[MeSH Terms] OR "Aerospace Medicine"[MeSH Terms] OR "Astronauts"[MeSH Terms] OR "space flight*"[Title/Abstract] OR "spaceflight*"[Title/Abstract] OR "Space Travel*"[Title/Abstract] OR "space explore*"[Title/Abstract] OR "Space Mission*"[Title/Abstract] OR "astronaut*"[Title/Abstract] OR "cosmonaut*"[Title/Abstract] OR "International Space Station"[Title/Abstract] OR "Mir Space Station"[Title/Abstract] OR "Long-duration spaceflight"[Title/Abstract]) OR (("Siberia"[MeSH Terms] OR "Extreme Environments"[MeSH Terms] OR "environment, controlled"[MeSH Terms] OR "Atlantic Ocean"[MeSH Terms] OR "Pacific Ocean"[MeSH Terms] OR "United States National Aeronautics and Space Administration"[MeSH Terms] OR "Seasons"[MeSH Terms] OR "Cold Climate"[MeSH Terms] OR "Cold Temperature"[MeSH Terms] OR "Darkness"[MeSH Terms] OR "Sunlight"[MeSH Terms] OR "Temperature"[MeSH Terms] OR "hypoxia/diet therapy"[MeSH Terms] OR "hypoxia/physiology"[MeSH Terms] OR "hypoxia/physiopathology"[MeSH Terms] OR "International Cooperation*"[MeSH Terms] OR "Workplace"[MeSH Terms] OR "Biomedical Research"[MeSH Terms] OR "Siberia"[Title/Abstract] OR "extreme environment*"[Title/Abstract] OR "extreme living condition*"[Title/Abstract] OR "Winterover"[Title/Abstract] OR "Winter-over"[Title/Abstract] OR "overwintering*"[Title/Abstract] OR "team member*"[Title/Abstract] OR "crew member*"[Title/Abstract] OR "research station*"[Title/Abstract] OR "simulation facility*"[Title/Abstract] OR "challenging habitat*"[Title/Abstract] OR "habitat research"[Title/Abstract] OR "human habitation*"[Title/Abstract] OR "Mars analog mission"[Title/Abstract] OR "Polar Research"[Title/Abstract] OR "Earth-based analogs"[Title/Abstract] OR "Ground analogs"[Title/Abstract] OR "NASA"[Title/Abstract] OR "Space Mission*"[Title/Abstract] OR "ISS"[Title/Abstract] OR "Euromir"[Title/Abstract] OR "Skylab"[Title/Abstract] OR "Space Administration"[Title/Abstract] OR "European Space Agency"[Title/Abstract] OR "ESA"[Title/Abstract] OR "season*"[Title/Abstract] OR "simulation condition*"[Title/Abstract] OR "Hypobaria"[Title/Abstract] OR "Hypoxia"[Title/Abstract] OR "high altitude"[Title/Abstract] OR "very low temperature"[Title/Abstract] OR "Extreme Medicine"[Title/Abstract] OR "Physiological System"[Title/Abstract] OR "Physiological Challenge"[Title/Abstract] OR "biomedical research project*"[Title/Abstract]) AND ("Confined Spaces"[MeSH Terms] OR "Isolated Habitat*"[Title/Abstract] OR "Ecological Systems, Closed"[MeSH Terms] OR "Breadboard Project"[Title/Abstract] OR "Regenerative Life Support System*" [Title/Abstract] OR "Bioregenerative life-support*"[Title/Abstract] OR "Sealed Cabin Ecology"[Title/Abstract] OR "CELSS"[Title/Abstract] OR (("Bed Rest"[Mesh] OR "Bed Rest*"[Title/Abstract]) AND ("Longterm"[Title/Abstract] OR "Long Term"[Title/Abstract])) OR "Ground-based stud*"[Title/Abstract] OR "closed-chamber stud*"[Title/Abstract] OR "ICE-environment*"[Title/Abstract] OR "ICE-research*"[Title/Abstract] OR "ICE-condition*"[Title/Abstract] OR "ICE-stud*"[Title/Abstract] OR "Isolated environment*"[Title/Abstract] OR "Confinement*"[Title/Abstract] OR "Confined environment*"[Title/Abstract] OR "Confinement condition*"[Title/Abstract] OR "Confined condition*"[Title/Abstract] OR "Indoor Workplace*"[Title/Abstract] OR "Isolation at sea"[Title/Abstract] OR "Closed environment*"[Title/Abstract] OR "Closed Ecological System*"[Title/Abstract] OR "Semi-closed environment*"[Title/Abstract] OR "Isolation period*"[Title/Abstract] OR "Limited antigen*"[Title/Abstract] OR "Limited antimicrobial*"[Title/Abstract] OR "Human adapti*"[Title/Abstract] OR "Winter-over syndrome"[Title/Abstract] OR "Polar T3 Syndrome"[Title/Abstract] OR "subsyndromal seasonal affective disorder*"[Title/Abstract]))) AND (("immunology"[MeSH Subheading] OR "Immune System"[Mesh] OR "Immune System Phenomena"[Mesh] OR "Cytokines"[Mesh] OR "Immunoproteins"[Mesh] OR Immunolog*[Title/Abstract] OR Cytokine*[Title/Abstract])) NOT ("Animals"[MeSH Terms] NOT ("Humans"[MeSH Terms] OR Human*[Title/Abstract]))

**Cochrane Library** (22.11.2022)	(((Antarcti* OR Arctic* OR (Space near/3 Simulation*) OR Ships OR Moon OR Mars OR Spacecraft OR (Extraterrestrial near/2 Environment*) OR "Biosphere 2" OR Envihab OR "SFINCSS-99" OR "Concordia Station" OR "Zhongshan Station" OR "Neumayer" OR "Maitri Station" OR "Great Wall Station" OR "Davis Station" OR "McMurdo Station" OR "Palmer Station" OR "South Pole" OR Arctowski OR "Vostok Station" OR "Syowa Station" OR "HI-SEAS" OR "Polar institute*" OR AGBRESA OR SIRIUS OR "Space Station*" OR Apollo OR "Lunar Palace" OR Submarine* OR "Land-based simulator*" OR "Space condition*" OR "Long-duration space exploration mission*" OR (Polar near/2 expedition*)):ti,ab,kw OR ((Space near/2 (Flight* OR Travel* OR Explor*)) OR Spaceflight* OR (Aerospace near/2 Medicine) OR Astronaut* OR Cosmonaut* OR "International Space Station" OR (Mir near/3 Space Station) OR ("Long-duration" near/2 spaceflight)):ti,ab,kw OR ((Siberia* OR (Extreme near/2 Environment*) OR "Atlantic Ocean" OR "Pacific Ocean" OR "United States National Aeronautics and Space Administration" OR (Biomedical near/3 Research) OR (Extreme near/3 "living condition*") OR Winterover OR Winter-over OR Overwintering* OR (Research near/2 Station*) OR (Simulation near/2 (Facilit* OR condition*)) OR "Challenging habitat*" OR (habitat near/2 research) OR "Human Habitation*" OR (Polar near/2 Research) OR "Earth-based analogs" OR "Ground analogs" OR NASA OR "European Space Agency" OR ESA OR Euromir OR Skylab OR (Space near/2 Mission) OR Season* OR Hypobaria OR Hypoxia OR "high altitude" OR "very low temperature*" OR "Extreme Medicine" OR (Physiological near/2 (System* OR Challenge*)) OR (Biomedical near/2 "research project*")):ti,ab,kw AND ((Confined near/2 (Space* OR environment* OR condition*)) OR (Closed near/3 "Ecological System*") OR (Semiclosed near/2 environment*) OR "Ground-based study" OR "Ground-based studies" OR "closed-chamber study" OR "closed-chamber studies" OR "ICE-environment*" OR "ICE-research" OR "ICE-condition*" OR "ICE-studies" OR "ICE-study" OR ((Isolated OR Closed) near/2 Environment*) OR Confinement* OR (Isolation near/5 Sea) OR (Isolation near/2 period*) OR "Human adaption" OR "Winter-over syndrome" OR "Polar T3 Syndrome" OR "Subsyndromal seasonal affective disorder*"):ti,ab,kw)) AND (Immunolog* OR Immunoprotein* OR Immunoglobulin* OR "Immune System" OR Immunit* OR Cytokine* OR Interleukin* OR TNF OR "Tumor Necrosis Factor*" OR TLR* OR "Toll-like Receptor*" OR Interferon* OR NF-kappa* OR Antigen* OR Chemokine* OR "C-reactive Protein*" OR Antibod* OR Leukocyte* OR Neutrophil* OR Macrophage*):ti,ab,kw) NOT (([mh "Animals"] OR animal*:ti,ab,kw) NOT ([mh "Humans"] OR human*:ti,ab,kw))
(28.01.2025, *old search term adapted to new Cochrane criteria*)	(((Antarcti* OR Arctic* OR (Space near/3 Simulation*) OR Ships OR Moon OR Mars OR Spacecraft OR (Extraterrestrial near/2 Environment*) OR "Biosphere 2" OR Envihab OR "SFINCSS-99" OR "Concordia Station" OR "Zhongshan Station" OR "Neumayer" OR "Maitri Station" OR "Great Wall Station" OR "Davis Station" OR "McMurdo Station" OR "Palmer Station" OR "South Pole" OR Arctowski OR "Vostok Station" OR "Syowa Station" OR "HI-SEAS" OR (Polar NEXT institute*) OR AGBRESA OR SIRIUS OR (Space NEXT Station*) OR Apollo OR "Lunar Palace" OR Submarine* OR (Land-based NEXT simulator*) OR (Space NEXT condition*) OR ("Long-duration space exploration" NEXT mission*) OR (Polar near/2 expedition*)):ti,ab,kw OR ((Space near/2 (Flight* OR Travel* OR Explor*)) OR Spaceflight* OR (Aerospace near/2 Medicine) OR Astronaut* OR Cosmonaut* OR "International Space Station" OR (Mir near/3 Space Station) OR ("Long-duration" near/2 spaceflight)):ti,ab,kw OR ((Siberia* OR (Extreme near/2 Environment*) OR "Atlantic Ocean" OR "Pacific Ocean" OR "United States National Aeronautics and Space Administration" OR (Biomedical near/3 Research) OR (Extreme near/3 "living condition") OR (Extreme near/3 "living conditions") OR Winterover OR Winter-over OR Overwintering* OR (Research near/2 Station*) OR (Simulation near/2 (Facilit* OR condition*)) OR (Challenging NEXT habitat*) OR (habitat near/2 research) OR (Human NEXT Habitation*) OR (Polar near/2 Research) OR "Earth-based analogs" OR "Ground analogs" OR NASA OR "European Space Agency" OR ESA OR Euromir OR Skylab OR (Space near/2 Mission) OR Season* OR Hypobaria OR Hypoxia OR "high altitude" OR "very low temperature" OR "very low temperatures" OR "Extreme Medicine" OR (Physiological near/2 (System* OR Challenge*)) OR (Biomedical near/2 (research NEXT project*))):ti,ab,kw AND ((Confined near/2 (Space* OR environment* OR condition*)) OR (Closed near/3 (Ecological NEXT System*)) OR (Semiclosed near/2 environment*) OR "Ground-based study" OR "Ground-based studies" OR "closed-chamber study" OR "closed-chamber studies" OR ICE-environment* OR ICE-research OR ICE-condition* OR ICE-studies OR ICE-study OR ((Isolated OR Closed) near/2 Environment*) OR Confinement* OR (Isolation near/5 Sea) OR (Isolation near/2 period*) OR "Human adaption" OR "Winter-over syndrome" OR "Polar T3 Syndrome" OR "Subsyndromal seasonal affective disorder" OR "Subsyndromal seasonal affective disorders"):ti,ab,kw)) AND (Immunolog* OR Immunoprotein* OR Immunoglobulin* OR "Immune System" OR Immunit* OR Cytokine* OR Interleukin* OR TNF OR "Tumor Necrosis Factor" OR "Tumor Necrosis Factors" OR TLR* OR "Toll-like Receptor" OR "Toll-like Receptors" OR Interferon* OR NF-kappa* OR Antigen* OR Chemokine* OR (C-reactive NEXT Protein*) OR Antibod* OR Leukocyte* OR Neutrophil* OR Macrophage*):ti,ab,kw) NOT (([mh "Animals"] OR animal*:ti,ab,kw) NOT ([mh "Humans"] OR human*:ti,ab,kw))
**Web of Science** (22.11.2022; 28.01.2025)	TS=(((((Antarcti* OR Arctic* OR "Space Simulation*" OR Ships OR Moon OR Mars OR Spacecraft OR "Extraterrestrial Environments" OR "Biosphere 2" OR Envihab OR "SFINCSS-99" OR "Concordia Station" OR "Zhongshan Station" OR "Neumayer" OR "Maitri Station" OR "Great Wall Station" OR "Davis Station" OR "McMurdo Station" OR "Palmer Station" OR "South Pole" OR Arctowski OR "Vostok Station" OR "Syowa Station" OR "HI-SEAS" OR "Polar institute*" OR AGBRESA OR SIRIUS OR "Space Station*"

OR Apollo OR "Lunar Palace" OR Submarine* OR "Land-based simulator*" OR "Space condition*" OR "Long-duration space exploration mission*" OR "Polar expedition*") NOT ("Sirius red stain*" OR "Sirius Trial*")) OR ("Space Flight*" OR Spaceflight* OR "Aerospace Medicine" OR "Space Travel*" OR "Space Explore*" OR Astronaut* OR Cosmonaut* OR "International Space Station" OR "Mir Space Station" OR ("Long-duration" NEAR/2 spaceflight)) OR ((Siberia* OR "Extreme Environment*" OR "Atlantic Ocean" OR "Pacific Ocean" OR "United States National Aeronautics and Space Administration" OR "Biomedical Research" OR "Extreme living condition*" OR Winterover OR Winter-over OR Overwintering* OR "Research Station*" OR "Simulation Facilit*" OR "Challenging habitat*" OR (habitat NEAR/2 research) OR "Human Habitation*" OR (Polar NEAR/2 Research) OR "Earth-based analogs" OR "Ground analogs" OR NASA OR "European Space Agency" OR ESA OR Euromir OR Skylab OR (Space NEAR/2 Mission) OR Season* OR "Simulation condition*" OR Hypobaria OR Hypoxia OR "high altitude" OR "very low temperature*" OR "Extreme Medicine" OR (Physiological NEAR/0 (System* OR Challenge*)) OR (Biomedical NEAR/2 "research project*")) AND ((Confined NEAR/2 (Space* OR environment* OR condition*)) OR "Closed Ecological System*" OR "Semiclosed environment*" OR "Ground-based study" OR "Ground-based studies" OR "closed-chamber study" OR "closed-chamber studies" OR "ICE-environment*" OR "ICE-research" OR "ICE-condition*" OR "ICE-studies" OR "ICE-study" OR "Isolated environment*" OR Confinement* OR (Isolation NEAR/5 sea) OR "Closed environment*" OR "isolation period*" OR "Human adaption" OR "Winter-over syndrome" OR "Polar T3 Syndrome" OR "Subsyndromal seasonal affective disorder*"))) AND (Immunolog* OR Immunoprotein* OR Immunoglobulin* OR "Immune System" OR Immunit* OR Cytokine* OR Interleukin* OR TNF OR "Tumor Necrosis Factor*" OR TLR* OR "Toll-like Receptor*" OR Interferon* OR NF-kappa* OR Antigen* OR Chemokine* OR "C-reactive Protein*" OR Antibod* OR Leukocyte* OR Neutrophil* OR Macrophage*)) NOT (Animal* NOT Human*))

Supplementary Table S1: Percentage of studies reporting differences (↑/↓) or constants (↔) over time across all habitats.

		All studies (n = 140)									Group 1: Space missions (n = 49)								
		During (n = 117)			Post (n = 82)			Pre/Post (n = 99)			During (n = 30)			Post (n = 35)			Pre/Post (n = 44)		
		↑	↓	↔	↑	↓	↔	↑	↓	↔	↑	↓	↔	↑	↓	↔	↑	↓	↔
myeloic cell line	Leu	7.7	23.3	16.7	6.1	6.1	1.2	3.0	1.0	8.1	6.7	3.3	3.3	2.9	8.6		2.3	2.3	4.5
	Mo	12.0	5.1	8.5	6.1	3.7	7.3	7.1	2.0	12.1	10.0		3.3	2.9	2.9	5.7	11.4	2.3	11.4
	Gra	8.5	4.3	2.6	2.4	8.5	2.4	5.1		11.1	10.0				11.4		9.1		9.1
	Eos	5.1		4.3	1.2		1.2			2.0				2.9					2.3
	Baso	2.6		0.9	1.2														0.0
	Neu	9.4	7.7	4.3	6.1	3.7	1.2	4.0	2.0	6.1	6.7				5.7				4.5
	Lys			0.9	3.7	1.2	1.2	3.0	2.0	1.0			3.3	8.6	2.9	2.9	6.8	4.5	2.3
	Phag	1.7	3.4			2.4									5.7				
lymphoid cell line	NK	6.8	12.8	5.1	8.5	19.5	3.7	4.0	9.1	12.1	3.3	16.7		8.6	20.0		2.3	11.4	9.1
	Lymp	12.8	5.1	8.5	2.4	9.8	8.5	3.0	2.0	11.1	3.3		3.3	2.9	2.9	5.7			9.1
	T	7.7	12.0	12.8	18.3	13.4	8.5	7.1	12.1	25.3	13.3	16.7	3.3	20.0	20.0	5.7	6.8	13.6	29.5
	B	5.1	2.6	11.1	2.4	7.3	8.5	4.0	1.0	17.2	10.0	3.3	6.7		11.4	5.7	4.5		18.2
immunglobuline	Ig		1.7					1.0	4.0	1.0							2.3	6.8	2.3
	IgA	10.3	4.3	8.5	7.3	7.3	3.7	2.0	2.0	13.1	6.7	3.3	3.3	11.4	8.6	5.7	2.3	2.3	13.6
	IgD			1.7			2.4			2.0			6.7			5.7			4.5
	IgE	2.6	0.9	6.0	1.2	1.2	6.1	1.0	1.0	9.1			6.7			5.7			9.1
	IgG	6.8	5.1	11.1	6.1	2.4	8.5	2.0	2.0	13.1		3.3	10.0	8.6	2.9	11.4	2.3		18.2
	IgM	6.0	4.3	12.0	2.4	4.9	4.9	2.0	1.0	14.1	3.3	3.3	13.3	5.7	2.9	8.6	4.5		20.5
factors of complement system	C1	0.9						1.0	1.0	1.0							2.3	2.3	
	C3	0.9	0.9	0.9		1.2				3.0					2.9				2.3
	C4	0.9	1.7	0.9						1.0									
	C5a	0.9		0.9															
	C8γ	0.9																	
	FD	0.9																	
	FH	0.9		0.9															
cytokines – proinflammatory	Cy	0.9	0.9	0.9			1.2	2.0	1.0	2.0		3.3	3.3			2.9	2.3	2.3	4.5
	IL-2	3.4	5.1	9.4	6.1	4.9	7.3	1.0	2.0	14.1	3.3	3.3	10.0	5.7		8.6	2.3	2.3	11.4
	IL-2a			0.9		1.2			1.0										
	IL-3	0.9		1.7		1.2				1.0	3.3				2.9				2.3
	IL-5		0.9	4.3	1.2		3.7			4.0		3.3	6.7	2.9		5.7			6.8
	IL-7	0.9		2.6		1.2	1.2			2.0	3.3				2.9				2.3
	IL-8	2.6		5.1	1.2	1.2	4.9	1.0		5.1	3.3		10.0	2.9	2.9	8.6	2.3		9.1
	IL-12	0.9	0.9	1.7	1.2	1.2	1.2		1.0	2.0	3.3				2.9				2.3
	IL-12p40	0.9		3.4		1.2	2.4			3.0	3.3		3.3		2.9	2.9			4.5
	IL-12p70	0.9		2.6	1.2		1.2	1.0		1.0	3.3		3.3	2.9		2.9	2.3		2.3
	IL-15	0.9		1.7		1.2				1.0	3.3				2.9	0.0			2.3
	IL-17			1.7			1.2			1.0			3.3			2.9			2.3
	IL-17a		2.6	1.7	2.4		1.2			3.0		3.3	3.3	2.9		2.9			4.5
	IL-18	1.7				1.2	1.2			1.0						2.9			2.3
	INF-α	1.7	1.7	0.9		2.4			1.0	2.0	6.7	3.3			5.7			2.3	4.5
	INF-γ	9.4	9.4	8.5	9.8	9.8	7.3	3.0	3.0	21.2	13.3	6.7	10.0	8.6	8.6	8.6	4.5	2.3	18.2
	TNF	1.7				1.2				2.0	6.7				2.9				4.5
	TNF-α	4.3	1.7	11.1	4.9	3.7	8.5	2.0	3.0	11.1			10.0	2.9	2.9	8.6		2.3	9.1
	ENA-78	0.9			1.2			1.0											
	MIP-1α	0.9	0.9	2.6	1.2			1.0											
	MIP-1β		0.9	1.7			1.2			1.0			3.3			2.9			2.3
	IP-10		0.9	0.9															
	Eotaxin		0.9	1.7															
cytokines – anti	IL-1Ra	4.3	3.4	2.6	2.4	8.5	1.2	3.0	2.0	3.0	13.3		3.3		14.3		2.3	2.3	6.8
	IL-4	0.9	1.7	7.7	2.4	2.4	8.5		1.0	11.1	3.3	3.3	6.7	2.9	2.9	5.7			13.6
	EGF	0.9		1.7															
cytokines – pro- & antiinflammatory	IL-1	1.7	0.9		1.2	3.7		1.0	1.0	3.0	6.7			2.9	5.7		2.3		6.8
	IL-1α	1.7	0.9	1.7		2.4				2.0	6.7				5.7				4.5
	IL-1β	2.6	2.6	8.5	1.2	6.1	6.1	1.0	2.0	8.1	6.7		3.3		5.7	2.9			6.8
	IL-6	2.6	3.4	10.3	4.9	4.9	8.5	2.0	3.0	12.1			13.3	2.9	2.9	11.4	4.5	2.3	9.1
	IL-10	5.1	5.1	7.7	7.3	2.4	7.3	3.0	2.0	11.1	6.7	3.3	10.0	5.7	2.9	11.4	4.5		15.9
	IL-13			3.4		1.2	2.4			2.0			3.3		2.9	2.9			2.3
	TNF-β		1.7	2.6	1.2		1.2			2.0			3.3		0.0	2.9			2.3
	TGF-β	0.9	3.4	2.6	1.2	2.4	3.7	1.0	1.0	4.0	3.3				2.9				2.3
	TGF-β1	1.7				1.2				2.0	3.3				2.9				2.3
	TGF-β2	0.9				1.2				1.0	3.3				2.9				2.3
	RANTES	5.1	0.9	1.7	1.2	2.4	1.2	2.0		1.0	6.7				5.7		2.3		2.3
	MCP-1	0.9	1.7	2.6	2.4			1.0		1.0			3.3	2.9					2.3
	MCP-1α																		
	G(M)-CSF	0.9	0.9	2.6		1.2	1.2	1.0		1.0									
	VEGF	0.9	1.7	0.9															
other factors	SAA		0.9				1.2			1.0									
	IGF-1	0.9				1.2				1.0	3.3				2.9				2.3
	Throm	0.9																	

		Group 2: Gravity simulation studies (n = 25)									Group 3: Terrestrial artificial habitat (n = 23)									Group 4: Terrestrial natural habitat (n = 46)								
		During (n = 23)			Post (n = 20)			Pre/Post (n = 19)			During (n = 19)			Post (n = 12)			Pre/Post (n = 16)			During (n = 45)			Post (n = 15)			Pre/Post (n = 20)		
		↑	↓	↔	↑	↓	↔	↑	↓	↔	↑	↓	↔	↑	↓	↔	↑	↓	↔	↑	↓	↔	↑	↓	↔	↑	↓	↔
myeloic cell line	Leu	4.3			5.0					10.5	10.5	10.5	5.3	8.3	8.3	8.3			12.5	8.9	8.9	6.7	13.3	6.7		10.0		10.0
	Mo	17.4	8.7	21.7	15.0	5.0	15.0	5.3	5.3	21.1	15.8	5.3	15.8			8.3	6.3		6.3	8.9	6.7	2.2	6.7	6.7				10.0
	Gra	8.7		8.7	5.0		10.0			15.8	10.5	10.5	5.3		8.3		6.3		6.3	6.7	6.7		6.7	13.3				15.0
	Eos	8.7		8.7			5.0			5.3	5.3									6.7		6.7						
	Baso	8.7		4.3	5.0					0.0	5.3																	
	Neu	13.0	4.3	4.3	5.0	5.0	5.0	5.3		10.5	10.5	10.5	5.3	16.7			12.5		6.3	8.9	13.3	6.7	13.3			5.0	10.0	5.0
	Lys																											
	Phag										5.3	5.3								2.2	6.7							
lymphoid cell line	NK	21.7	17.4		10.0	25.0	5.0	10.5	15.8	5.3	5.3	26.3	26.3	8.3	33.3	8.3		6.3	37.5	2.2	2.2	2.2	6.7		6.7	5.0		5.0
	Lymp	17.4	13.0	21.7	5.0	15.0	25.0	10.5	5.3	21.1	31.6	5.3	5.3		16.7		6.3	0.0	6.3	8.9	4.4	6.7		13.3			5.0	10.0
	T	8.7	21.7	21.7	30.0	10.0	10.0	15.8	10.5	31.6	15.8	10.5	31.6	16.7	16.7	8.3	6.3	12.5	31.3		4.4	6.7			13.3		10.0	5.0
	B	4.3	4.3	17.4		5.0	15.0	5.3		15.8	5.3	5.3	21.1	16.7	0.0	8.3			31.3	2.2		6.7		6.7	6.7	5.0	5.0	5.0
immunglobuline	Ig																				4.4						5.0	
	IgA	13.0		4.3	5.0	5.0				5.3		5.3	21.1		8.3	8.3			25.0	15.6	6.7	4.4	6.7	6.7		5.0	5.0	10.0
	IgD																											
	IgE	4.3									5.3		10.5			8.3		6.3	18.8	2.2	2.2	6.7	6.7	6.7	13.3	5.0		10.0
	IgG	13.0	4.3	8.7	10.0		10.0	5.3		5.3		5.3	15.8			8.3		6.3	18.8	11.1	6.7	11.1		6.7			5.0	5.0
	IgM	13.0				5.0			5.3			5.3	21.1		8.3	8.3			25.0	6.7	6.7	13.3		6.7				5.0
factors of complement system	C1																		6.3	2.2								
	C3																		6.3	2.2	2.2	2.2						5.0
	C4																			2.2	4.4	2.2						5.0
	C5a																			2.2		2.2						
	C8γ																			2.2								
	FD																			2.2								
	FH																			2.2		2.2						
cytokines – proinflammatory	Cy							5.3			5.3																	
	IL-2	4.3	13.0	13.0	10.0	5.0	10.0			26.3		10.5	21.1	8.3	16.7	8.3		6.3	18.8	4.4		2.2		6.7				5,0
	IL-2a																					2.2		6.7			5.0	
	IL-3												5.3									2.2						
	IL-5												10.5			8.3			6.3			2.2						
	IL-7												10.5			8.3			6.3			2.2						
	IL-8												10.5			8.3			6.3	4.4		2.2						
	IL-12												5.3			8.3			6.3		2.2	2.2	6.7				5.0	
	IL-12p40			4.3			5.0			5.3			5.3									2.2						
	IL-12p70												5.3									2.2						
	IL-15												5.3									2.2						
	IL-17																					2.2						
	IL-17a		8.7		5.0					5.3			5.3															
	IL-18	4.3				5.0					5.3																	
	INF-α											5.3										2.2						
	INF-γ	8.7	21.7	4.3	20.0	5.0	5.0	5.3	5.3	26.3	15.8	10.5	21.1	8.3	25.0	16.7		6.3	43.8	4.4	4.4	4.4		6.7				5,0
	TNF																											
	TNF-α	4.3		13.0	5.0	5.0	15.0	5.3	5.3	15.8	5.3		15.8	8.3	0.0	8.3		6.3	18.8	6.7	4.4	8.9	6.7	6.7		5.0		5,0
	ENA-78																			2.2			6.7			5.0		
	MIP-1α											5.3	5.3							2.2		4.4	6.7			5.0		
	MIP-1β												5.3								2.2							
	IP-10											5.3										2.2						
	Eotaxin											5.3	5.3									2.2						
cytokines – anti	IL-1Ra	4.3			5.0	5.0	5.0	5.3					5.3								8.9	2.2	6.7	6.7		5.0	5.0	
	IL-4		4.3	8.7	5.0		10.0		5.3	10.5			21.1		8.3	25.0			18.8			2.2						
	EGF										5.3		5.3									2.2						
cytokines – pro- & antiinflammatory	IL-1																				2.2			6.7			5.0	
	IL-1α											5.3	5.3									2.2						
	IL-1β	4.3	8.7	8.7	5.0	10.0	10.0		10.5	15.8		5.3	10.5		8.3	8.3	6.3		6.3			11.1			6.7			5,0
	IL-6	4.3	4.3	4.3	5.0	5.0	5.0			15.8		5.3	15.8	8.3		16.7		6.3	18.8	4.4	4.4	8.9	6.7	13.3			5.0	10,0
	IL-10	13.0		4.3	10.0	5.0	5.0	5.3		10.5	5.3	5.3	10.5			8.3		6.3	6.3		8.9	6.7	13.3				5.0	5,0
	IL-13												10.5			8.3			6.3			2.2						
	TNF-β		4.3		5.0					5.3		5.3	5.3						0.0			2.2						
	TGF-β												5.3			8.3			6.3		8.9	4.4	6.7	6.7	13.3	5.0	5.0	10,0
	TGF-β1	4.3								5.3																		
	TGF-β2																											
	RANTES	8.7	4.3	4.3	5.0		5.0	5.3			5.3		5.3							2.2								
	MCP-1		4.3		5.0			5.3			5.3	5.3										4.4						
	MCP-1α																											
	G(M)-CSF												10.5			8.3			6.3	2.2	2.2	2.2		6.7		5.0		
	VEGF										5.3		5.3								4.4							
other factors	SAA																				2.2				6.7			5.0
	IGF-1																											
	Throm										5.3																	

Notes: The percentage change in each parameter was evaluated across all habitats as well as within each specific habitat. 'x%' indicates the proportion of studies at that time point (pre/during [here: during], during/post [here: post], pre/post) within that habitat reporting the respective outcome. For clarity, "no reports" or 0.0% are represented by empty cells in the table. *Abbreviations:* anti: anti-inflammatory; B: B-cells; Baso: Basophils; C: Complement component; Cy: Cytokines; Eos: Eosinophils; EGF: Epidermal Growth Factor; ENA78: Epithelial Neutrophil Activating Peptide 78; Eotaxin: Eotaxin chemokine; FD: Factor D; FH: Factor H; G(M)CSF: Granulocyte (Macrophage) Colony-Stimulating Factor; Gra: Granulocytes; IGF: Insulin-like Growth Factor; Ig: Immunoglobulin; IL: Interleukin; INF: Interferon; IP10: Interferon gamma-induced protein 10; Leu: Leukocytes; Lymp: Lymphocytes; Lys: Lysozyme; MCP: Monocyte Chemoattractant Protein; MIP: Macrophage Inflammatory Protein; Mo: Monocytes; NK: Natural Killer cells; Neu: Neutrophils; Phag: Phagocytes; RANTES: Regulated on Activation, Normal T cell Expressed and Secreted; SAA: Serum Amyloid A; T: T-cells; TGF: Transforming Growth Factor; Throm: Thrombocytes; TNF: Tumor Necrosis Factor; VEGF: Vascular Endothelial Growth Factor.

Supplementary Table S2: Reported differences (↑/↓) or constants (↔) across all habitats in pre-/during comparison.

Group 1: Space missions

	ID	1	2	3	4	5	6	7	8	9	10	11	12	13, 14, 15	16	17	18	19	20	21	22	23	24	25	26	27	28	29	30	31	32	33	34	35	36	37	38	39	40	41	42	43	44	45	46	47, 48	49
myeolic cell line	Leu																	↑								↑							↓					↔									
	Mo					↔						↑														↑	↑																				
	Gra											↑														↑	↑																				
	Eos																																														
	Baso																																														
	Neu					↑												↑																													
	Lys																																								↔						
	Phag																																														
lymphoid cell line	NK				↓	↓						↑											↓					↓																		↓ ↓	
	Lymp																	↔								↑																					
	T					↑						↔		↑			↓						↓			↑		↑↓						↓												↓ ↓ ↓ ↓ ↓	
	B					↑						↑		↔												↑																		↔		↓	
immunoglobulins	Ig																																														
	IgA															↓	↔	↔			↔↑																						↑				
	IgD																↔	↔																													
	IgE																↔	↔																													
	IgG															↓	↔	↔																									↔				
	IgM	↔														↓	↔	↔			↑																						↔				
complement system	C1																																														
	C3																																														
	C4																																														
	C5a																																														
	C8G																																														
	FD																																														
	FH																																														
cytokines – Proinflammatory	Cy													↔																				↓													
	IL-2					↔					↔				↓															↔				↑													
	IL-2a																																														
	IL-3																								↑																						
	IL-5										↔	↓																		↔																	
	IL-7																								↑																						
	IL-8										↑															↔				↔	↔																
	IL-12																													↑																	
	IL-12p40																								↑					↔																	
	IL-12p70																													↔	↑																
	IL-15																								↑																						
	IL-17										↔																																				
	IL-17a											↓																		↔																	
	IL-18																									↔																					
	IFN-α												↓		↑															↑																	
	IFN-γ					↔					↔	↓	↑↓		↑											↔				↑				↑													
	TNF					↑							↑																																		
	TNF-α																									↔				↔	↔																
	ENA-78																																														
	MIP-1α																																														
	MIP-1β																														↔																
	IP-10																																														
	Eotaxin																																														
cytokines – anti	IL-1Ra					↑					↑		↑																	↑	↔																
	IL-4										↔	↓														↔				↑																	
	EGF																																														
cytokines – pro- and antiinflammatory	IL-1												↑																	↑																	
	IL-1α												↑																		↑																
	IL-1β					↑							↑													↔																					
	IL-6					↔																				↔				↔	↔																
	IL-10					↑					↔	↓														↔				↔				↑													
	IL-13																													↔																	
	TNF-β																													↔																	
	TGF-β					↑																																									
	TGF-β1																								↑																						
	TGF-β2																								↑																						
	RANTES										↑														↑																						
	MCP-1																														↔																
	MCP-1α																																														
	G(M)-CSF																																														
	VEGF																																														
other factors	SAA																																														
	IGF-1												↑																																		
	Throm																																														

Supplementary Table S2: Reported differences (↑/↓) or constants (↔) across all habitats in pre-/during comparison, following.

Group 2: Gravity simulation studies

	ID	50	51	52	53	54	55	56	57	58	59	60	61	62	63	64	65	66	67	68	69	70	71	72	73	74
myeolic cell line	Leu				↑																					
	Mo				↔↑	↔				↓		↑					↑	↔			↔	↔	↓		↑	
	Gra					↔			↔ / ↔	↑								↑								
	Eos				↔↑							↑									↔					
	Baso				↔↑							↑														
	Neu		↔		↑												↑				↓		↑			
	Lys																									
	Phag																									
lymphoid cell line	NK					↑				↑			↓				↑↓	↓				↓	↑		↑	
	Lymp	↔	↔		↑	↔						↑	↓			↔↑	↓	↔			↓		↑			
	T								↔ / ↔↑↓	↑			↓	↓			↓			↔↑		↔	↓		↔	↔
	B	↔								↔										↑		↔	↔		↓	
immunoglobulins	Ig																									
	IgA	↑									↑						↑							↔		
	IgD																									
	IgE																								↑	
	IgG	↔									↑↓						↑							↔↑		
	IgM	↑									↑						↑									
complement system	C1																									
	C3																									
	C4																									
	C5a																									
	C8G																									
	FD																									
	FH																									
cytokines	Cy																									
cytokines – proinflammatory	IL-2								↔ / ↔						↓		↔ / ↑					↓	↓			↔
	IL-2a																									
	IL-3																									
	IL-5																									
	IL-7																									
	IL-8																									
	IL-12																									
	IL-12p40																		↔ / ↔ / ↔							
	IL-12p70																									
	IL-15																									
	IL-17																									
	IL-17a																								↓	↓
	IL-18																								↑	
	IFN-α																									
	IFN-γ					↔			↑↓									↑				↓	↓		↓	↓
	TNF																									
	TNF-α			↔		↔												↔	↑ / ↑ / ↑							
	ENA-78																									
	MIP-1α																									
	MIP-1β																									
	IP-10																									
	Eotaxin																									
cytokines – anti	IL-1Ra																		↑ / ↑ / ↑							
	IL-4																					↓	↔			↔
	EGF																									
cytokines – pro- and antiinflammatory	IL-1																									
	IL-1α																									
	IL-1β			↔		↔												↓					↓		↑	
	IL-6			↔		↑																	↓			
	IL-10																	↑	↔ / ↔ / ↔			↑			↑	
	IL-13																									
	TNF-β																						↓			
	TGF-β																									
	TGF-β1																								↑	
	TGF-β2																									
	RANTES									↔									↓ / ↑ / ↑							
	MCP-1																								↓	
	MCP-1α																									
	G(M)-CSF																									
	VEGF																									
other factors	SAA																									
	IGF-1																									
	Throm																									

Group 3: Terrestrial artificial habitats

	ID	75	76	77	78	79	80	81	82	83	84	85	86	87	88	89	90	91	92	93	94	95	96	97
myeolic cell line	Leu							↑						↑↓				↔						↓
	Mo				↔	↔		↑				↑↓					↑		↔					
	Gra				↑	↔↓						↑↓												
	Eos							↑																
	Baso							↑																
	Neu							↑											↑	↓		↓	↔	
	Lys																							
	Phag											↑↓												
lymphoid cell line	NK	↓			↔	↔			↓			↔↓	↓				↑		↓			↔	↔	
	Lymp				↑	↔↑				↓		↑		↑			↑			↑				
	T	↑			↔	↔			↓			↔↓	↑						↔		↔	↑	↔	
	B				↔	↔							↓						↔			↑	↔	
immunoglobulins	Ig																							
	IgA		↔ / ↔						↓	↔		↔		↔										
	IgD																							
	IgE		↔ / ↔											↔↑										
	IgG		↔ / ↔						↓			↔		↔										
	IgM		↔ / ↔						↓	↔		↔		↔										
complement system	C1																							
	C3																							
	C4																							
	C5a																							
	C8G																							
	FD																							
	FH																							
cytokines	Cy											↑												
cytokines – proinflammatory	IL-2	↓				↔												↔	↓			↔	↔	
	IL-2a																							
	IL-3					↔																		
	IL-5					↔												↔						
	IL-7					↔												↔						
	IL-8					↔												↔						
	IL-12																	↔						
	IL-12p40					↔																		
	IL-12p70					↔																		
	IL-15					↔																		
	IL-17																							
	IL-17a					↔																		
	IL-18																		↑					
	IFN-α					↓																		
	IFN-γ			↑	↔	↔↓						↓					↑	↔				↑	↔	
	TNF																							
	TNF-α					↔												↔				↑	↔	
	ENA-78																							
	MIP-1α					↔↓																		
	MIP-1β					↔																		
	IP-10					↑↓																		
	Eotaxin					↔↓																		
cytokines – anti	IL-1Ra					↔																		
	IL-4		↔ / ↔			↔								↔				↔						
	EGF					↔↑																		
cytokines – pro- and antiinflammatory	IL-1																							
	IL-1α					↔↓																		
	IL-1β					↔												↔	↓					
	IL-6			↓	↔	↔												↔						
	IL-10					↔↓						↑						↔						
	IL-13					↔												↔						
	TNF-β					↔↓																		
	TGF-β																	↔						
	TGF-β1																							
	TGF-β2																							
	RANTES					↔↑																		
	MCP-1					↑↓																		
	MCP-1α																							
	G(M)-CSF					↔												↔						
	VEGF					↔↑																		
other factors	SAA																							
	IGF-1																							
	Throm							↑																

Supplementary Table S2: Reported differences (↑/↓) or constants (↔) across all habitats in pre-/during comparison, following.

Group 4: Terrestrial natural habitats

	ID	98	99	100	101		102	103	104	105		106	107	108	109	110		111			112	113	114	115	116	117	118	119
myeolic cell line	Leu							↔		↔						↔	↔											
	Mo									↑				↑											↑			
	Gra												↑	↑														
	Eos									↔															↑			
	Baso																											
	Neu									↔															↑			
	Lys																											
	Phag																							↑↓				
lymphoid cell line	NK													↓														
	Lymp									↔			↓															
	T													↓														↓
	B	↔												↑														
immunoglobulins	Ig																									↓		
	IgA						↓					↑				↔	↔	↔	↔	↔	↑	↑						
	IgD																											
	IgE														↑↓↔												↔	
	IgG		↔									↓				↔	↔	↔	↔	↓	↑	↑						
	IgM		↔									↓				↔	↔	↔	↔	↔		↑						
complement system	C1				↑	↑																						
	C3				↑	↑																						
	C4				↓	↑																						
	C5a				↔	↑																						
	C8G																											
	FD				↑	↑																						
	FH				↔	↑																						
cytokines	Cy																											
cytokines – proinflammatory	IL-2									↔	↔																	
	IL-2a								↔																			
	IL-3									↔																		
	IL-5									↔	↔																	
	IL-7									↔	↔																	
	IL-8			↑						↔	↔																	
	IL-12																											
	IL-12p40									↔																		
	IL-12p70									↔	↔																	
	IL-15									↔																		
	IL-17									↔																		
	IL-17a																											
	IL-18																											
	IFN-α									↔																		
	IFN-γ									↓	↔																	↑
	TNF																											
	TNF-α			↔						↓	↔																	↑
	ENA-78													↑														
	MIP-1α									↔																		
	MIP-1β									↓																		
	IP-10									↔																		
	Eotaxin									↔																		
cytokines – anti	IL-1Ra									↓				↓														
	IL-4									↔	↔																	
	EGF									↔																		
cytokines – pro- and antiinflammatory	IL-1																											
	IL-1α									↔																		
	IL-1β								↔	↔	↔																	
	IL-6			↑						↔	↔												↑					
	IL-10									↓	↔																	
	IL-13									↔	↔																	
	TNF-β									↔																		
	TGF-β						↓																					↓
	TGF-β1																											
	TGF-β2																											
	RANTES									↑																		
	MCP-1			↔						↔																		
	MCP-1α																											
	G(M)-CSF									↓	↔			↑														
	VEGF									↓																		
other factors	SAA																											
	IGF-1																											
	Throm																											

	ID	120	121	122	123	124	125	126	127	128	129	130	131	132	133	134	135	136	137	138		139	140	141	142	143
myeolic cell line	Leu							↓				↓				↑↓				↑	↑	↑	↑↓			
	Mo													↓	↓							↓	↑			↔
	Gra														↓					↑	↑	↓	↓			
	Eos																					↔↑	↑			↔
	Baso																									
	Neu							↓			↓	↑↓		↓								↑↓↔	↑↓			↔
	Lys																									
	Phag											↓				↓										
lymphoid cell line	NK																		↑					↔		
	Lymp							↑						↑	↓					↑	↑	↔↑				↔
	T									↔							↔							↔		
	B									↔														↔		
immunoglobulins	Ig											↓														
	IgA			↑									↑									↑↓			↑	↓
	IgD																									
	IgE																		↔							
	IgG			↓									↑				↔					↑			↔	↑
	IgM			↔									↑				↔					↑↓			↔	↓
complement system	C1																									
	C3									↔																↓
	C4									↔																↓
	C5a																									
	C8G										↑															
	FD																									
	FH																									
cytokines	Cy																									
cytokines – proinflammatory	IL-2																		↑					↑		
	IL-2a																									
	IL-3																									
	IL-5																									
	IL-7																									
	IL-8				↑																					
	IL-12										↓							↔								
	IL-12p40																									
	IL-12p70																									
	IL-15																									
	IL-17																									
	IL-17a																									
	IL-18																									
	IFN-α																									
	IFN-γ	↑																↔							↓	
	TNF																									
	TNF-α	↑			↔														↔					↓	↑	
	ENA-78																									
	MIP-1α	↔																								
	MIP-1β																								↑	
	IP-10																									
	Eotaxin																									
cytokines – anti	IL-1Ra																	↓	↔					↓		
	IL-4																									
	EGF																									
cytokines – pro- and antiinflammatory	IL-1																							↓		
	IL-1α																									
	IL-1β				↔		↔												↔							
	IL-6				↔		↔								↓				↔					↓		
	IL-10						↔				↓							↓	↔					↓		
	IL-13																									
	TNF-β																									
	TGF-β	↓				↔																		↔	↓	
	TGF-β1																									
	TGF-β2																									
	RANTES																									
	MCP-1																									
	MCP-1α																									
	G(M)-CSF																									
	VEGF				↓																					
other factors	SAA					↓																				
	IGF-1																									
	Throm																									

Notes: The table represent the direction of studies reporting an increase (↑), a decrease (↓) or no change in each immunological parameter (↔) for the comparison pre-/during-mission. The corresponding study for each ID is listed below. *Abbreviations:* anti: anti-inflammatory; B: B-cells; Baso: Basophils; C: Complement component; Cy: Cytokines; Eos: Eosinophils; EGF: Epidermal Growth Factor; ENA78: Epithelial Neutrophil Activating Peptide 78; Eotaxin: Eotaxin chemokine; FD: Factor D; FH: Factor H; G(M)-CSF: Granulocyte (Macrophage) Colony-Stimulating Factor; Gra: Granulocytes; IGF: Insulin-like Growth Factor; Ig: Immunoglobulin; IL: Interleukin; INF: Interferon; IP10: Interferon gamma-induced protein 10; Leu: Leukocytes; Lymp: Lymphocytes; Lys: Lysozyme; MCP: Monocyte Chemoattractant Protein; MIP: Macrophage Inflammatory Protein; Mo: Monocytes; NK: Natural Killer cells; Neu: Neutrophils; Phag: Phagocytes; RANTES: Regulated on Activation, Normal T cell Expressed and Secreted; SAA: Serum Amyloid A; T: T-cells; TGF: Transforming Growth Factor; Throm: Thrombocytes; TNF: Tumor Necrosis Factor; VEGF: Vascular Endothelial Growth Factor.

1 Agha et al. (2019)
2 Benjamin et al. (2016)
3 Berendeeva et al. (2015)
4 Bigley et al. (2019)
5 Buchheim et al. (2019)
6 Buchheim et al. (2020)
7 Burakova et al. (2007)
8 Capri et al. (2019)
9 Crucian et al. (2008)
10 Crucian et al. (2014)
11 Crucian et al. (2015)
12 DaSilveira et al. (2020)
13 Garett-Bakelmann et al. (2019)
14 Bezdan et al. (2020)
15 Gertz et al. (2020)
16 Grigoriev et al. (1991)
17 Guseva and Tashpulatov (1979)
18 Kimzey (1975)
19 Kimzey (1975)
20 Kimzey et al. (1976)
21 Konstantinova et al. (1978)
22 Konstantinova et al. (1985)
23 Konstantinova (1991)
24 Konstantinova et al. (1991)
25 Konstantinova et al. (1995)
26 Krieger et al. (2021)
27 Kuzichkin et al. (2022)
28 Kuzichkin et al. (2023)
29 Lesniak et al. (1998)
30 Manie et al. (1991)
31 Mehta et al. (2022)
32 Mehta et al. (20242)
33 Meshkov and Rykova (1995)
34 Meshkov et al. (1998)
35 Morukov et al. (2010)
36 Morukov et al. (2011)
37 Nikolaeva et al. (1982)
38 Pastushkova et al. (2021)
39 Poliakov and Noskov (2005)
40 Ponomarev et al. (2016)
41 Rykova et al. (2006)
42 Rykova et al. (2008)
43 Spielmann et al. (2018)
44 Spielmann et al. (2019)
45 Stahn et al. (2017)
46 Stowe et al. (2011)
47 Vorobyov et al. (1983)
48 Vorob´ev et al. (1984)
49 Vorob´ev et al. (1986)
50 Bonnefoy et al. (2022)
51 Jacob et al. (2022)
52 Brooks et al. (2014)
53 Buescher et al. (2024)
54 Chouker et al. (2001)
55 Clement et al. (2022)
56 Clement et al. (2022)
57 Crucian et al. (2009)
58 Hoff et al. (2014)
59 Ivanova et al. (2005)
60 Kalandarova (1991)
61 Lesniak et al. (1998)
62 Lesniak et al. (1999)
63 Meshkov et al. (1998)
64 Novoderzhkina et al. (1996)
65 Schmitt et al. (1996)
66 Schmitt et al. (2000)
67 Shearer et al. (2009)
68 Sonnenfeld et al. (2007)
69 Trudel et al. (2009)
70 Uchakin et al. (2002)
71 Uchakin et al. (2007)
72 Volozhin et al. (2001)
73 Xu et al. (2013)
74 Xu et al. (2016)
75 Antropova et al. (2000)
76 Burakova et al. (2007)
77 Chen et al. (2020)
78 Chouker et al. (2002)
79 Douglas et al. (2022)
80 Hao et al. (2022)
81 Kalandarova et al. (1983)
82 Konstantinova et al. (1997)
83 Larkin et al. (1972)
84 Li et al. (2022)
85 Morukov et al. (2013)
86 Nwanaji-Enwerem et al. (2020)
87 Rykova et al. (2004)
88 Schmitt et al. (1995)
89 Husson (1996)
90 Sonnenfeld et al. (1992)
91 Strewe et al. (2015)
92 Uchakin et al. (2006)
93 Walford et al. (1992)
94 Xun et al. (2018)
95 Yi et al. (2014)
96 Yi et al. (2015)
97 Yuan et al. (2019)
98 Allen et al. (1973)
99 Bell et al. (1987)
100 Bhushan et al. (2019)
101 Bhushan et al. (2021a)
102 Bhushan et al. (2021b)
103 Cameron et al. (1968)
104 Chen et al. (2016)
105 Diak et al. (2024)
106 Evdokimov et al. (1983)
107 Feuerecker et al. (2014)
108 Feuerecker et al. (2019)
109 Feuerecker et al. (2022)
110 Flynn et al. (1977)
111 Gleeson et al. (2000)
112 Hammermeister et al. (1992)
113 Holmes et al. (1971)
114 Johnsen et al. (2021)
115 Kantorovich (1970)
116 Kovardakov et al. (1976)
117 Kurbanov et al. (1977)
118 Lund and Dowdle (1977)
119 Metha et al. (2000)
120 Mishra et al. (2010)
121 Mishra et al. (2011)
122 Mishra et al. (2012)
123 Moraes et al. (2023)
124 Moraes et al. (2024)
125 Mrakic-Sposta et al. (2022)
126 Muchmore et al. (1970)
127 Muchmore and Shurley (1974)
128 Muller et al. (1995)
129 Nieman et al. (2020)
130 Novikov et al. (1991)
131 Roberts-Thomson et al. (1985)
132 Ryabinin (1972)
133 Sakai et al. (2004)
134 Sapov et al. (1981)
135 Shearer et al. (2001)
136 Shearer et al. (2002)
137 Shirai et al. (2003)
138 Strewe et al. (2019)
139 Tashpulatov et al. (1971)
140 Tashpulatov et al. (1976)
141 Tingate et al. (1997)
142 Yadav et al. (2012)
143 Žákovská (2023)

Supplementary Table S3: Reported differences (↑/↓) or constants (↔) across all habitats in during-/post comparison.

Group 1: Space missions

	ID	1	2	3	4	5	6	7	8	9	10	11	12	13, 14, 15	16	17	18	19	20	21	22	23	24	25	26	27	28	29	30	31	32	33	34	35	36	37	38	39	40	41	42	43	44	45	46	47, 48	49
myeolic cell line	Leu																	↓								↓							↓	↑													
	Mo					↔				↔		↓														↔								↑											↓		
	Gra					↓				↓		↓														↓								↑													
	Eos																																												↑		
	Baso																																														
	Neu																	↓																											↓		
	Lys															↑		↑																	↑↓							↔					
	Phag																																	↓	↓												
lymphoid cell line	NK				↓	↑				↑		↓								↓			↑					↓						↓												↓	↓
	Lymp																	↔								↓								↑											↔		
	T		↑			↓				↔		↔		↓			↑	↑	↑	↑↓		↑						↓						↑↓	↓											↓	↓
	B					↓				↓		↓		↔																				↓										↔			
immunoglobu-lins	Ig																																														
	IgA															↑	↔	↔	↑	↑↓															↑↓								↓				
	IgD																↔	↔																													
	IgE																↔	↔																													
	IgG	↔															↔	↔	↑	↑↓															↑								↔				
	IgM																↔	↔																	↑↓								↔				↑
complement system	C1																																														
	C3																	↓																													
	C4																																														
	C5a																																														
	C8G																																														
	FD																																														
	FH																																														
cytokines	Cy													↔																																	
cytokines – proinflammatory	IL-2					↔					↔				↑															↔				↑													
	IL-2a																																														
	IL-3																								↓																						
	IL-5										↔	↑																		↔																	
	IL-7																								↓																						
	IL-8										↓																↔			↔	↔			↑													
	IL-12																													↓																	
	IL-12p40																								↓					↔																	
	IL-12p70																													↔	↑																
	IL-15																								↓																						
	IL-17										↔																																				
	IL-17a											↑																		↔																	
	IL-18																										↔																				
	IFN-α														↓															↓																	
	IFN-γ					↔				↑	↔	↑			↓												↔			↓				↓											↑		
	TNF					↓																																									
	TNF-α																										↔			↔	↔			↓											↑		
	ENA-78																																														
	MIP-1α																																														
	MIP-1β																														↔																
	IP-10																																														
	Eotaxin																																														
cytokines – anti	IL-1Ra					↓					↓		↓																	↓	↓																
	IL-4										↔	↑															↔			↓																	
	EGF																																														
cytokines – pro- and antiinflammatory	IL-1												↓																	↓				↑													
	IL-1α												↓																		↓																
	IL-1β					↓							↓														↔																				
	IL-6					↔																					↔			↔	↔			↑↓													
	IL-10					↓				↔	↔	↑															↔			↔				↑													
	IL-13																													↔				↓													
	TNF-β																													↔																	
	TGF-β					↓																																									
	TGF-β1																								↓																						
	TGF-β2																								↓																						
	RANTES										↓														↓																						
	MCP-1																														↑																
	MCP-1α																																														
	G(M)-CSF																																														
	VEGF																																														
other factors	SAA																																														
	IGF-1												↓																																		
	Throm																																														

Supplementary Table S3: Reported differences (↑/↓) or constants (↔) across all habitats in during-/post comparison, following.

Group 2: Gravity simulation studies

	ID	50	51	52	53	54	55	56	57		58	59	60	61	62	63	64	65		66	67			68	69	70	71	72	73	74
myeolic cell line	Leu															↑														
	Mo					↔							↑							↔					↑	↔	↑		↓	
	Gra					↔			↔	↔										↓										
	Eos																								↔					
	Baso												↑																	
	Neu		↔																						↑		↓			
	Lys																													
	Phag																													
lymphoid cell line	NK					↓								↓					↑	↔↓						↑	↓		↓	
	Lymp	↔	↔			↔								↓			↔↓			↔					↑		↓			
	T								↔	↔↑				↑↓	↑				↓							↔	↑		↑	↑
	B	↔																								↔	↔		↓	
immunoglobu-lins	Ig																													
	IgA	↓										↑																		
	IgD																													
	IgE																													
	IgG	↔										↑																↔↑		
	IgM	↓																												
complement sys-tem	C1																													
	C3																													
	C4																													
	C5a																													
	C8G																													
	FD																													
	FH																													
cytokines	Cy																													
cytokines – proinflammatory	IL-2								↔	↔									↓							↑	↑			↔
	IL-2a																													
	IL-3																													
	IL-5																													
	IL-7																													
	IL-8																													
	IL-12																													
	IL-12p40																				↔	↔	↔							
	IL-12p70																													
	IL-15																													
	IL-17																													
	IL-17a																													↑
	IL-18																												↓	
	IFN-α																													
	IFN-γ					↔				↑										↓						↑	↑			↑
	TNF																													
	TNF-α			↔		↔														↔	↑	↓	↓							
	ENA-78																													
	MIP-1α																													
	MIP-1β																													
	IP-10																													
	Eotaxin																													
cytokines – anti	IL-1Ra																				↔	↓	↑↓							
	IL-4																									↑	↔			↔
	EGF																													
cytokines – pro- and antiinflammatory	IL-1																													
	IL-1α																													
	IL-1β			↔		↔														↓							↑		↓	
	IL-6			↔		↓																					↑			
	IL-10																			↓	↔	↔	↔			↑			↑	
	IL-13																													
	TNF-β																										↑			
	TGF-β																													
	TGF-β1																													
	TGF-β2																													
	RANTES																				↑	↔	↑							
	MCP-1																												↑	
	MCP-1α																													
	G(M)-CSF																													
	VEGF																													
other factors	SAA																													
	IGF-1																													
	Throm																													

Group 3: Terrestrial artificial habitats

	ID	75	76		77	78		79	80	81	82	83	84	85	86	87	88	89	90	91	92	93	94	95	96	97
myeolic cell line	Leu															↓				↔						↑
	Mo						↔																			
	Gra						↓																			
	Eos																									
	Baso																									
	Neu																				↑				↑	
	Lys																									
	Phag																									
lymphoid cell line	NK	↓					↔				↓				↓				↓						↑	
	Lymp						↓												↓							
	T	↑					↔				↓				↓										↑	
	B						↔								↑										↑	
immunoglobu-lins	Ig																									
	IgA		↔	↔							↓															
	IgD																									
	IgE		↔	↔																						
	IgG		↔	↔																						
	IgM		↔	↔							↓															
complement sys-tem	C1																									
	C3																									
	C4																									
	C5a																									
	C8G																									
	FD																									
	FH																									
cytokines	Cy																									
cytokines – proinflammatory	IL-2	↓																		↔	↓				↑	
	IL-2a																									
	IL-3																									
	IL-5																			↔						
	IL-7																			↔						
	IL-8																			↔						
	IL-12																			↔						
	IL-12p40																									
	IL-12p70																									
	IL-15																									
	IL-17																									
	IL-17a																									
	IL-18																									
	IFN-α																									
	IFN-γ				↓		↔												↓	↔	↓				↑	
	TNF																									
	TNF-α																			↔					↑	
	ENA-78																									
	MIP-1α																									
	MIP-1β																									
	IP-10																									
	Eotaxin																									
cytokines – anti	IL-1Ra																									
	IL-4		↔	↔												↔				↔	↓					
	EGF																									
cytokines – pro- and antiinflammatory	IL-1																									
	IL-1α																									
	IL-1β																			↔	↓					
	IL-6				↑		↔													↔						
	IL-10																			↔						
	IL-13																			↔						
	TNF-β																									
	TGF-β																			↔						
	TGF-β1																									
	TGF-β2																									
	RANTES																									
	MCP-1																									
	MCP-1α																									
	G(M)-CSF																									
	VEGF																									
other factors	SAA																									
	IGF-1																									
	Throm																									

Supplementary Table S3: Reported differences (↑/↓) or constants (↔) across all habitats in during-/post comparison, following.

Group 4: Terrestrial natural habitats

	ID	98	99	100	101	102	103	104	105	106	107	108	109	110	111	112	113	114	115	116	117	118	119	120
myeolic cell line	Leu																							
	Mo											↓												
	Gra											↓												
	Eos																							
	Baso																							
	Neu																							
	Lys																							
	Phag																							
lymphoid cell line	NK											↑												
	Lymp																							
	T											↔												
	B											↓												
immunoglobulins	Ig																							
	IgA					↑																		
	IgD																							
	IgE												↑↓↔									↔		
	IgG																							
	IgM																							
complement system	C1																							
	C3																							
	C4																							
	C5a																							
	C8G																							
	FD																							
	FH																							
cytokines	Cy																							
cytokines: proinflammatory	IL-2																							
	IL-2a							↓																
	IL-3																							
	IL-5																							
	IL-7																							
	IL-8																							
	IL-12																							
	IL-12p40																							
	IL-12p70																							
	IL-15																							
	IL-17																							
	IL-17a																							
	IL-18																							
	IFN-α																							
	IFN-γ																							↓
	TNF																							
	TNF-α																							↓
	ENA-78											↑												
	MIP-1α																							↑
	MIP-1β																							
	IP-10																							
	Eotaxin																							
cytokines: Anti	IL-1Ra											↓												
	IL-4																							
	EGF																							
cytokines: pro- and antiinflammatory	IL-1																							
	IL-1α																							
	IL-1β							↔																
	IL-6																	↓						
	IL-10																							
	IL-13																							
	TNF-β																							
	TGF-β					↑																		↓
	TGF-β1																							
	TGF-β2																							
	RANTES																							
	MCP-1																							
	MCP-1α																							
	G(M)-CSF																							
	VEGF																							
other factors	SAA																							
	IGF-1																							
	Throm																							

	ID	121	122	123	124	125	126	127	128	129	130	131	132	133	134	135	136	137	138	139	140	141	142	143
myeolic cell line	Leu						↑												↓ ↑					
	Mo													↑										
	Gra													↑					↓ ↓					
	Eos																							
	Baso																							
	Neu						↑			↑														
	Lys																							
	Phag																							
lymphoid cell line	NK																					↔		
	Lymp						↓												↓ ↓					
	T																					↔		
	B																					↔		
immunoglobulins	Ig																							
	IgA											↓												
	IgD																							
	IgE																							
	IgG											↓												
	IgM											↓												
complement system	C1																							
	C3																							
	C4																							
	C5a																							
	C8G																							
	FD																							
	FH																							
cytokines	Cy																							
cytokines: proinflammatory	IL-2																					↓		
	IL-2a																							
	IL-3																							
	IL-5																							
	IL-7																							
	IL-8																							
	IL-12									↑														
	IL-12p40																							
	IL-12p70																							
	IL-15																							
	IL-17																							
	IL-17a																							
	IL-18																							
	IFN-α																							
	IFN-γ																							
	TNF																							
	TNF-α																					↑		
	ENA-78																							
	MIP-1α																							
	MIP-1β																							
	IP-10																							
	Eotaxin																							
cytokines: Anti	IL-1Ra																					↑		
	IL-4																							
	EGF																							
cytokines: pro- and antiinflammatory	IL-1																					↓		
	IL-1α																							
	IL-1β																							
	IL-6													↑								↓		
	IL-10									↑												↑		
	IL-13																							
	TNF-β																							
	TGF-β				↔																	↔		
	TGF-β1																							
	TGF-β2																							
	RANTES																							
	MCP-1																							
	MCP-1α																							
	G(M)-CSF																							
	VEGF																							
other factors	SAA				↔																			
	IGF-1																							
	Throm																							

Notes: The table represent the direction of studies reporting an increase (↑), a decrease (↓) or no change in each immunological parameter (↔) for the comparison during-/post-mission. The corresponding study for each ID is listed below. *Abbreviations*: anti: anti-inflammatory; B: B-cells; Baso: Basophils; C: Complement component; Cy: Cytokines; Eos: Eosinophils; EGF: Epidermal Growth Factor; ENA78: Epithelial Neutrophil Activating Peptide 78; Eotaxin: Eotaxin chemokine; FD: Factor D; FH: Factor H; G(M)-CSF: Granulocyte (Macrophage) Colony-Stimulating Factor; Gra: Granulocytes; IGF: Insulin-like Growth Factor; Ig: Immunoglobulin; IL: Interleukin; INF: Interferon; IP10: Interferon gamma-induced protein 10; Leu: Leukocytes; Lymp: Lymphocytes; Lys: Lysozyme; MCP: Monocyte Chemoattractant Protein; MIP: Macrophage Inflammatory Protein; Mo: Monocytes; NK: Natural Killer cells; Neu: Neutrophils; Phag: Phagocytes; RANTES: Regulated on Activation, Normal T cell Expressed and Secreted; SAA: Serum Amyloid A; T: T-cells; TGF: Transforming Growth Factor; Throm: Thrombocytes; TNF: Tumor Necrosis Factor; VEGF: Vascular Endothelial Growth Factor.

1 Agha et al. (2019)
2 Benjamin et al. (2016)
3 Berendeeva et al. (2015)
4 Bigley et al. (2019)
5 Buchheim et al. (2019)
6 Buchheim et al. (2020)
7 Burakova et al. (2007)
8 Capri et al. (2019)
9 Crucian et al. (2008)
10 Crucian et al. (2014)
11 Crucian et al. (2015)
12 DaSilveira et al. (2020)
13 Garett-Bakelmann et al. (2019)
14 Bezdan et al. (2020)
15 Gertz et al. (2020)
16 Grigoriev et al. (1991)
17 Guseva and Tashpulatov (1979)
18 Kimzey (1975)
19 Kimzey (1975)
20 Kimzey et al. (1976)
21 Konstantinova et al. (1978)
22 Konstantinova et al. (1985)
23 Konstantinova (1991)
24 Konstantinova et al. (1991)
25 Konstantinova et al. (1995)
26 Krieger et al. (2021)
27 Kuzichkin et al. (2022)
28 Kuzichkin et al. (2023)
29 Lesniak et al. (1998)
30 Manie et al. (1991)
31 Mehta et al. (2022)
32 Mehta et al. (20242)
33 Meshkov and Rykova (1995)
34 Meshkov et al. (1998)
35 Morukov et al. (2010)
36 Morukov et al. (2011)
37 Nikolaeva et al. (1982)
38 Pastushkova et al. (2021)
39 Poliakov and Noskov (2005)
40 Ponomarev et al. (2016)
41 Rykova et al. (2006)
42 Rykova et al. (2008)
43 Spielmann et al. (2018)
44 Spielmann et al. (2019)
45 Stahn et al. (2017)
46 Stowe et al. (2011)
47 Vorobyov et al. (1983)
48 Vorob´ev et al. (1984)
49 Vorob´ev et al. (1986)
50 Bonnefoy et al. (2022)
51 Jacob et al. (2022)
52 Brooks et al. (2014)
53 Buescher et al. (2024)
54 Chouker et al. (2001)
55 Clement et al. (2022)
56 Clement et al. (2022)
57 Crucian et al. (2009)
58 Hoff et al. (2014)
59 Ivanova et al. (2005)
60 Kalandarova (1991)
61 Lesniak et al. (1998)
62 Lesniak et al. (1999)
63 Meshkov et al. (1998)
64 Novoderzhkina et al. (1996)
65 Schmitt et al. (1996)
66 Schmitt et al. (2000)
67 Shearer et al. (2009)
68 Sonnenfeld et al. (2007)
69 Trudel et al. (2009)
70 Uchakin et al. (2002)
71 Uchakin et al. (2007)
72 Volozhin et al. (2001)
73 Xu et al. (2013)
74 Xu et al. (2016)
75 Antropova et al. (2000)
76 Burakova et al. (2007)
77 Chen et al. (2020)
78 Chouker et al. (2002)
79 Douglas et al. (2022)
80 Hao et al. (2022)
81 Kalandarova et al. (1983)
82 Konstantinova et al. (1997)
83 Larkin et al. (1972)
84 Li et al. (2022)
85 Morukov et al. (2013)
86 Nwanaji-Enwerem et al. (2020)
87 Rykova et al. (2004)
88 Schmitt et al. (1995)
89 Husson (1996)
90 Sonnenfeld et al. (1992)
91 Strewe et al. (2015)
92 Uchakin et al. (2006)
93 Walford et al. (1992)
94 Xun et al. (2018)
95 Yi et al. (2014)
96 Yi et al. (2015)
97 Yuan et al. (2019)
98 Allen et al. (1973)
99 Bell et al. (1987)
100 Bhushan et al. (2019)
101 Bhushan et al. (2021a)
102 Bhushan et al. (2021b)
103 Cameron et al. (1968)
104 Chen et al. (2016)
105 Diak et al. (2024)
106 Evdokimov et al. (1983)
107 Feuerecker et al. (2014)
108 Feuerecker et al. (2019)
109 Feuerecker et al. (2022)
110 Flynn et al. (1977)
111 Gleeson et al. (2000)
112 Hammermeister et al. (1992)
113 Holmes et al. (1971)
114 Johnsen et al. (2021)
115 Kantorovich (1970)
116 Kovardakov et al. (1976)
117 Kurbanov et al. (1977)
118 Lund and Dowdle (1977)
119 Metha et al. (2000)
120 Mishra et al. (2010)
121 Mishra et al. (2011)
122 Mishra et al. (2012)
123 Moraes et al. (2023)
124 Moraes et al. (2024)
125 Mrakic-Sposta et al. (2022)
126 Muchmore et al. (1970)
127 Muchmore and Shurley (1974)
128 Muller et al. (1995)
129 Nieman et al. (2020)
130 Novikov et al. (1991)
131 Roberts-Thomson et al. (1985)
132 Ryabinin (1972)
133 Sakai et al. (2004)
134 Sapov et al. (1981)
135 Shearer et al. (2001)
136 Shearer et al. (2002)
137 Shirai et al. (2003)
138 Strewe et al. (2019)
139 Tashpulatov et al. (1971)
140 Tashpulatov et al. (1976)
141 Tingate et al. (1997)
142 Yadav et al. (2012)
143 Žákovská (2023)

Supplementary Table S4: Reported differences (↑/↓) or constants (↔) across all habitats in pre-/post comparison.

Group 1: Space missions

	ID	1	2	3	4	5	6	7	8	9	10	11	12	13	14	15	16	17	18	19	20	21	22	23	24		25	26	27	28	29	30		31	32	33	34	35	36	37	38		39	40	41	42	43	44	45	46	47	48	49
myeolic cell line	Leu																			↔									↑									↓	↔															
	Mo			↔↓		↔				↔		↑																	↑										↔	↑					↑		↑				↔			
	Gra					↔				↔		↔																	↑										↔	↑					↑		↑							
	Eos																																																↔			
	Baso																																																			
	Neu																			↔																													↔			
	Lys																	↑↓	↑	↑																				↓								↔						
	Phag																																						↓															
lymphoid cell line	NK				↓	↔				↔		↔												↓	↑		↔		↓							↓										↓							
	Lymp																						↔						↔									↔												↔			
	T		↔			↔				↔		↔		↔			↔↓		↔	↑		↔↓		↓					↑		↔↓	↑↓	↔	↓				↔	↔							↔							
	B					↔	↑			↔		↔		↔								↔	↔						↑																	↔			↔				
immunoglobu-lins	Ig													↓									↔																	↓	↓	↑											
	IgA							↔										↔↑	↔	↔		↓																							↔			↔					
	IgD																		↔	↔																																	
	IgE							↔											↔	↔																									↔								
	IgG	↔						↔										↔↑	↔	↔		↔																							↔			↔					
	IgM							↔										↔↑	↔	↔		↔	↔																		↔	↑			↔			↔					
complement system	C1																																																			
	C3																			↔																																	
	C4																																																				
	C5a																																																				
	C8G																																																				
	FD																																																				
	FH																																																				
cytokinesG	Cy													↔																								↔	↓							↑							
proinflammatory	IL-2					↔					↔						↔							↓			↔					↑		↔																			
	IL-2a																																																				
	IL-3																											↔																									
	IL-5										↔	↔																						↔																			
	IL-7																											↔																									
	IL-8										↔																		↔					↔	↔			↑															
	IL-12																																	↔																			
	IL-12p40																											↔						↔																			
	IL-12p70																																	↔	↑																		
	IL-15																											↔																									
	IL-17										↔																																										
	IL-17a											↔																						↔																			
	IL-18																												↔																								
	IFN-α																↔									↓								↔																			
	IFN-γ					↔				↔	↔	↔					↔									↑			↔					↔				↑↓												↔			
	TNF					↔																					↔																										
	TNF-α																												↔					↔	↔			↓												↔			
	ENA-78																																																				
	MIP-1a																																																				
	MIP-1β																																		↔																		
	IP-10																																																				
	Eotaxin																																																				
anti	IL-1Ra					↓					↑		↔																					↔	↔																		
	IL-4							↔			↔	↔																	↔					↔											↔								
	EGF																																																				
pro- and antiinflammatory	IL-1												↔																			↔	↔	↔				↑															
	IL-1α												↔																						↔																		
	IL-1β					↔							↔																↔																								
	IL-6					↔			↑																				↔					↔	↔			↑↓															
	IL-10					↔				↔	↔	↔																	↔					↔				↑												↔↑			
	IL-13																																	↔				↓															
	TNF-β																																	↔																			
	TGF-β					↔																																															
	TGF-β1																											↔																									
	TGF-β2																											↔																									
	RANTES										↑																	↔																									
	MCP-1																																		↔																		
	MCP-1α																																																				
	G(M)-CSF																																																				
	VEGF																																																				
other factors	SAA																																																				
	IGF-1												↔																																								
	Throm																																																				

Supplementary Table S4: Reported differences (↑/↓) or constants (↔) across all habitats in pre-/post comparison, following.

Group 2: Gravity simulation studies

	ID	50	51	52	53	54	55			56	57		58	59	60	61	62	63	64	65	66	67			68	69	70	71	72	73	74
myeolic cell line	Leu													↔				↔													
	Mo					↔	↓	↔	↔												↔					↑	↔	↔			
	Gra					↔					↔	↔									↔										
	Eos																									↔					
	Baso																														
	Neu		↔																							↑		↔			
	Lys																														
	Phag																														
lymphoid cell line	NK					↑										↓				↔↑	↓						↓				
	Lymp	↔	↔			↔				↑						↓					↔					↑					
	T									↑	↔	↔↑				↑↓	↔			↓					↔		↔	↔			↔
	B	↔								↑																	↔	↔			
immunoglobulins	Ig																														
	IgA	↔																													
	IgD																														
	IgE																														
	IgG	↔								↑																					
	IgM	↓																													
complement system	C1																														
	C3																														
	C4																														
	C5a																														
	C8G																														
	FD																														
	FH																														
cytokines	Cy									↑																					
cytokines – proinflammatory	IL-2										↔	↔								↔							↔	↔			↔
	IL-2a																														
	IL-3																														
	IL-5																														
	IL-7																														
	IL-8																														
	IL-12																														
	IL-12p40																					↔	↔	↔							
	IL-12p70																														
	IL-15																														
	IL-17																														
	IL-17a																														↔
	IL-18																														
	IFN-α																														
	IFN-γ					↔						↔↑									↔						↓	↔			↔
	TNF																														
	TNF-α			↔		↔															↔	↑	↓	↑							
	ENA-78																														
	MIP-1a																														
	MIP-1β																														
	IP-10																														
	Eotaxin																														
cytokines – anti	IL-1Ra																					↑	↑	↑							
	IL-4																										↓	↔			↔
	EGF																														
cytokines – pro- and antiinflammatory	IL-1																														
	IL-1α																														
	IL-1β			↔		↔															↓							↔		↓	
	IL-6			↔		↔																						↔			
	IL-10																				↔	↔	↔	↔			↑				
	IL-13																														
	TNF-β																											↔			
	TGF-β																														
	TGF-β1																													↔	
	TGF-β2																														
	RANTES																					↑	↑	↑							
	MCP-1																													↑	
	MCP-1α																														
	G(M)-CSF																														
	VEGF																														
other factors	SAA																														
	IGF-1																														
	Throm																														

Group 3: Terrestrial artificial habitats

	ID	75	76	77	78		79	80	81	82	83	84	85	86	87	88	89	90	91	92	93	94	95	96	97
myeolic cell line	Leu																		↔						↔
	Mo					↔						↑													
	Gra				↑	↔																			
	Eos																								
	Baso																								
	Neu				↑															↑				↔	
	Lys																								
	Phag																								
lymphoid cell line	NK	↔			↔	↔				↔				↓		↔		↔						↔	
	Lymp				↑	↑												↔							
	T	↑			↔	↓				↔		↓		↔		↔								↔	
	B	↔			↔	↔								↔		↔								↔	
immunoglobulins	Ig																								
	IgA	↔	↔							↔						↔									
	IgD																								
	IgE		↔												↔↓	↔									
	IgG	↓	↔							↔						↔									
	IgM	↔	↔							↔						↔									
complement system	C1																								
	C3															↔									
	C4															↔									
	C5a																								
	C8G																								
	FD																								
	FH																								
cytokines	Cy																								
cytokines – proinflammatory	IL-2	↓														↔			↔					↔	
	IL-2a																								
	IL-3																								
	IL-5																		↔						
	IL-7																		↔						
	IL-8																		↔						
	IL-12																		↔						
	IL-12p40																								
	IL-12p70																								
	IL-15																								
	IL-17																								
	IL-17a																								
	IL-18																								
	IFN-α																								
	IFN-γ			↔	↔	↔		↔								↔		↔	↔	↓				↔	
	TNF																								
	TNF-α							↓								↔			↔					↔	
	ENA-78																								
	MIP-1a																								
	MIP-1β																								
	IP-10																								
	Eotaxin																								
cytokines – anti	IL-1Ra																								
	IL-4		↔												↔				↔						
	EGF																								
cytokines – pro- and antiinflammatory	IL-1																								
	IL-1α																								
	IL-1β							↑											↔						
	IL-6			↔	↔	↔		↓											↔						
	IL-10							↓											↔						
	IL-13																		↔						
	TNF-β																								
	TGF-β																		↔						
	TGF-β1																								
	TGF-β2																								
	RANTES																								
	MCP-1																								
	MCP-1α																								
	G(M)-CSF																		↔						
	VEGF																								
other factors	SAA																								
	IGF-1																								
	Throm																								

Supplementary Table S4: Reported differences (↑/↓) or constants (↔) across all habitats in pre-/post comparison, following.

Group 4: Terrestrial natural habitats

	ID	98	99	100	101	102	103	104	105	106	107	108	109	110	111	112	113	114	115	116	117	118	119	120	121	122	123	124	125	126	127	128	129	130	131	132	133	134	135	136	137	138		139	140	141	142	143
myeolic cell line	Leu																			↑										↔												↔	↑					
	Mo											↔																									↔											
	Gra											↔																									↔					↔	↔					
	Eos																																															
	Baso																																															
	Neu																													↑	↓		↓		↔													
	Lys																																															
	Phag																																															
lymphoid cell line	NK											↑																																		↔		
	Lymp																													↓					↔							↔	↔					
	T											↓																						↓												↔		
	B											↑																						↓												↔		
immunoglobulins	Ig									↓																																						
	IgA					↑				↔																								↓	↔													
	IgD																																															
	IgE												↔↑									↔																										
	IgG																																	↓	↔													
	IgM																																		↔													
complement system	C1																																															
	C3																																		↔													
	C4																																		↔													
	C5a																																															
	C8G																																															
	FD																																															
	FH																																															
cytokines	Cy																																															
proinflammatory	IL-2																																													↔		
	IL-2a							↓																																								
	IL-3																																															
	IL-5																																															
	IL-7																																															
	IL-8																																															
	IL-12																																↓															
	IL-12p40																																															
	IL-12p70																																															
	IL-15																																															
	IL-17																																															
	IL-17a																																															
	IL-18																																															
	IFN-α																																															
	IFN-γ																							↔																								
	TNF																																															
	TNF-α																							↑																						↔		
	ENA-78											↑																																				
	MIP-1a																							↑																								
	MIP-1β																																															
	IP-10																																															
	Eotaxin																																															
anti	IL-1Ra											↓																																		↑		
	IL-4																																															
	EGF																																															
pro- and antiinflammatory	IL-1																																													↓		
	IL-1α																																															
	IL-1β							↔																																								
	IL-6																	↔																			↔									↓		
	IL-10																																↓													↔		
	IL-13																																															
	TNF-β																																															
	TGF-β					↑																		↓				↔																		↔		
	TGF-β1																																															
	TGF-β2																																															
	RANTES																																															
	MCP-1																																															
	MCP-1α																																															
	G(M)-CSF											↑																																				
	VEGF																																															
other factors	SAA																											↔																				
	IGF-1																																															
	Throm																																															

Notes: The table represent the direction of studies reporting an increase (↑), a decrease (↓) or no change in each immunological parameter (↔) for the comparison pre-/post-mission. The corresponding study for each ID is listed below. *Abbreviations*: anti: anti-inflammatory; B: B-cells; Baso: Basophils; C: Complement component; Cy: Cytokines; Eos: Eosinophils; EGF: Epidermal Growth Factor; ENA78: Epithelial Neutrophil Activating Peptide 78; Eotaxin: Eotaxin chemokine; FD: Factor D; FH: Factor H; G(M)-CSF: Granulocyte (Macrophage) Colony-Stimulating Factor; Gra: Granulocytes; IGF: Insulin-like Growth Factor; Ig: Immunoglobulin; IL: Interleukin; INF: Interferon; IP10: Interferon gamma-induced protein 10; Leu: Leukocytes; Lymp: Lymphocytes; Lys: Lysozyme; MCP: Monocyte Chemoattractant Protein; MIP: Macrophage Inflammatory Protein; Mo: Monocytes; NK: Natural Killer cells; Neu: Neutrophils; Phag: Phagocytes; RANTES: Regulated on Activation, Normal T cell Expressed and Secreted; SAA: Serum Amyloid A; T: T-cells; TGF: Transforming Growth Factor; Throm: Thrombocytes; TNF: Tumor Necrosis Factor; VEGF: Vascular Endothelial Growth Factor.

1 Agha et al. (2019)
2 Benjamin et al. (2016)
3 Berendeeva et al. (2015)
4 Bigley et al. (2019)
5 Buchheim et al. (2019)
6 Buchheim et al. (2020)
7 Burakova et al. (2007)
8 Capri et al. (2019)
9 Crucian et al. (2008)
10 Crucian et al. (2014)
11 Crucian et al. (2015)
12 DaSilveira et al. (2020)
13 Garett-Bakelmann et al. (2019)
14 Bezdan et al. (2020)
15 Gertz et al. (2020)
16 Grigoriev et al. (1991)
17 Guseva and Tashpulatov (1979)
18 Kimzey (1975)
19 Kimzey (1975)
20 Kimzey et al. (1976)
21 Konstantinova et al. (1978)
22 Konstantinova et al. (1985)
23 Konstantinova (1991)
24 Konstantinova et al. (1991)
25 Konstantinova et al. (1995)
26 Krieger et al. (2021)
27 Kuzichkin et al. (2022)
28 Kuzichkin et al. (2023)
29 Lesniak et al. (1998)
30 Manie et al. (1991)
31 Mehta et al. (2022)
32 Mehta et al. (20242)
33 Meshkov and Rykova (1995)
34 Meshkov et al. (1998)
35 Morukov et al. (2010)
36 Morukov et al. (2011)
37 Nikolaeva et al. (1982)
38 Pastushkova et al. (2021)
39 Poliakov and Noskov (2005)
40 Ponomarev et al. (2016)
41 Rykova et al. (2006)
42 Rykova et al. (2008)
43 Spielmann et al. (2018)
44 Spielmann et al. (2019)
45 Stahn et al. (2017)
46 Stowe et al. (2011)
47 Vorobyov et al. (1983)
48 Vorob´ev et al. (1984)
49 Vorob´ev et al. (1986)
50 Bonnefoy et al. (2022)
51 Jacob et al. (2022)
52 Brooks et al. (2014)
53 Buescher et al. (2024)
54 Chouker et al. (2001)
55 Clement et al. (2022)
56 Clement et al. (2022)
57 Crucian et al. (2009)
58 Hoff et al. (2014)
59 Ivanova et al. (2005)
60 Kalandarova (1991)
61 Lesniak et al. (1998)
62 Lesniak et al. (1999)
63 Meshkov et al. (1998)
64 Novoderzhkina et al. (1996)
65 Schmitt et al. (1996)
66 Schmitt et al. (2000)
67 Shearer et al. (2009)
68 Sonnenfeld et al. (2007)
69 Trudel et al. (2009)
70 Uchakin et al. (2002)
71 Uchakin et al. (2007)
72 Volozhin et al. (2001)
73 Xu et al. (2013)
74 Xu et al. (2016)
75 Antropova et al. (2000)
76 Burakova et al. (2007)
77 Chen et al. (2020)
78 Chouker et al. (2002)
79 Douglas et al. (2022)
80 Hao et al. (2022)
81 Kalandarova et al. (1983)
82 Konstantinova et al. (1997)
83 Larkin et al. (1972)
84 Li et al. (2022)
85 Morukov et al. (2013)
86 Nwanaji-Enwerem et al. (2020)
87 Rykova et al. (2004)
88 Schmitt et al. (1995)
89 Husson (1996)
90 Sonnenfeld et al. (1992)
91 Strewe et al. (2015)
92 Uchakin et al. (2006)
93 Walford et al. (1992)
94 Xun et al. (2018)
95 Yi et al. (2014)
96 Yi et al. (2015)
97 Yuan et al. (2019)
98 Allen et al. (1973)
99 Bell et al. (1987)
100 Bhushan et al. (2019)
101 Bhushan et al. (2021a)
102 Bhushan et al. (2021b)
103 Cameron et al. (1968)
104 Chen et al. (2016)
105 Diak et al. (2024)
106 Evdokimov et al. (1983)
107 Feuerecker et al. (2014)
108 Feuerecker et al. (2019)
109 Feuerecker et al. (2022)
110 Flynn et al. (1977)
111 Gleeson et al. (2000)
112 Hammermeister et al. (1992)
113 Holmes et al. (1971)
114 Johnsen et al. (2021)
115 Kantorovich (1970)
116 Kovardakov et al. (1976)
117 Kurbanov et al. (1977)
118 Lund and Dowdle (1977)
119 Metha et al. (2000)
120 Mishra et al. (2010)
121 Mishra et al. (2011)
122 Mishra et al. (2012)
123 Moraes et al. (2023)
124 Moraes et al. (2024)
125 Mrakic-Sposta et al. (2022)
126 Muchmore et al. (1970)
127 Muchmore and Shurley (1974)
128 Muller et al. (1995)
129 Nieman et al. (2020)
130 Novikov et al. (1991)
131 Roberts-Thomson et al. (1985)
132 Ryabinin (1972)
133 Sakai et al. (2004)
134 Sapov et al. (1981)
135 Shearer et al. (2001)
136 Shearer et al. (2002)
137 Shirai et al. (2003)
138 Strewe et al. (2019)
139 Tashpulatov et al. (1971)
140 Tashpulatov et al. (1976)
141 Tingate et al. (1997)
142 Yadav et al. (2012)
143 Žákovská (2023)

Supplementary Figure S1: Risk of bias analysis, traffic lights.

Study	Bias due to confounding	Bias in selection of participants into the study	Bias in classification of interventions	Bias due to deviations from intended interventions	Bias due to missing data	Bias in measurement of outcomes	Bias in selection of the reported result	Overall Risk of Bias
Agha et al., 2020	!	✓	✓	✓	✓	✓	✓	!
Allen et al., 1973	!	✓	✓	✓	✓	✓	✓	!
Antropova et al., 2020	!	✓	✓	✓	✓	✓	✓	!
Bell et al., 1987	!	✓	✓	✓	✓	✓	✓	!
Benjam et al., 2016	!	✓	✓	✓	✓	✓	✓	!
Berendeeva et al., 2015	!	✓	✓	✓	✓	✓	✓	!
Bezdan et al., 2020	✓	✓	✓	✓	✓	✓	✓	✓
Bhusan et al., 2021a	✓	✓	✓	✓	✓	✓	✓	✓
Bhusan et al., 2021b	!	✓	✓	✓	✓	✓	✓	!
Bhushan et al., 2019	!	✓	✓	✓	✓	✓	✓	!
Bigley et al., 2019	!	✓	✓	✓	✓	✓	✓	!
Bonnefoy et al., 2022	✓	✓	✓	✓	✓	✓	✓	✓
Brooks et al., 2014	✓	✓	✓	✓	✓	✓	✓	✓
Buchheim et al., 2019	!	✓	✓	✓	✓	✓	✓	!
Buchheim et al., 2020	✓	✓	✓	✓	✓	✓	✓	✓
Buescher et al., 2024	✓	✓	✓	✓	✓	✓	✓	✓
Buravkova et al., 2007	!	✓	✓	✓	✓	✓	✓	!
Cameron et al., 1968	!	✓	✓	✓	✓	✓	✓	!
Capri et al., 2019	✓	✓	✓	✓	✓	✓	✓	✓
Chen et al., 2016	!	✓	✓	✓	✓	✓	✓	!
Chen et al., 2020	!	✓	✓	✓	✓	✓	✓	!
Chouker et al., 2001	!	✓	✓	✓	✓	✓	✓	!
Chouker et al., 2002	✓	✓	✓	✓	✓	✓	✓	✓
Clement et al., 2022a	!	✓	✓	✓	✓	✓	✓	!
Clement et al., 2022b	!	✓	✓	✓	✓	✓	✓	!
Crucian et al., 2008	!	✓	✓	✓	✓	✓	✓	!
Crucian et al., 2009	!	✓	✓	!	!	✓	✓	!
Crucian et al., 2014	!	✓	✓	✓	✓	✓	✓	!
Crucian et al., 2015	!	✓	✓	✓	✓	✓	✓	!
daSilveira et al., 2020	✓	✓	✓	✓	✓	✓	✓	✓
Diak et al., 2024	✓	✓	✓	✓	!	✓	✓	!
Douglas et al., 2022	✓	✓	✓	✓	✓	✓	✓	✓
Evdokimov et al., 1983	!	✓	✓	✓	✓	✓	✓	!
Feuerecker et al., 2014	✓	✓	✓	✓	✓	✓	✓	✓
Feuerecker et al., 2019	✓	✓	✓	✓	✓	✓	✓	✓
Feuerecker et al., 2020	✓	✓	✓	✓	✓	✓	✓	✓
Flynn et al., 1976	!	✓	✓	✓	✓	✓	✓	!
Garrett-Bakelman et al., 2020	!	✓	✓	✓	✓	✓	✓	!
Gertz et al., 2020	!	✓	✓	✓	✓	✓	✓	!
Gleeson et al., 2000	!	✓	✓	✓	✓	✓	✓	!
Grigoriev et al., 1991	!	✓	✓	✓	✓	✓	✓	!
Guseva et al., 1979	!	✓	✓	✓	✓	✓	✓	!
Hammermeister et al., 1992	✓	✓	✓	✓	✓	✓	✓	✓
Hao et al., 2022	!	✓	✓	✓	✓	✓	✓	!
Hoff et al., 2015	!	✓	✓	✓	✓	✓	✓	!
Holmes et al., 1970	!	✓	✓	!	✓	✓	✓	!
Husson et al., 1996	!	✓	✓	✓	✓	✓	✓	!
Ivanova et al., 2005	!	✓	✓	✓	✓	✓	✓	!
Jacob et al., 2022	✓	✓	✓	✓	✓	✓	✓	✓
Johnson et al., 2021	✗	✓	✓	✓	✓	✓	✓	✗
Kalandarova et al., 1983	!	✓	✓	✓	✓	✓	✓	!
Kalandarova et al., 1991	!	✓	✓	✓	✓	✓	✓	!
Kantorovich et al., 1970	!	✓	✓	✓	✓	✓	✓	!
Kimzey et al., 1975	!	✓	✓	✓	✓	✓	✓	!
Kimzey et al., 1976	!	✓	✓	✓	✓	✓	✓	!
Konstantinova et al., 1978	!	✓	✓	✓	✓	✓	✓	!
Konstantinova et al., 1985	!	✓	✓	✓	✓	✓	✓	!
Konstantinova et al., 1991	!	✓	✓	✓	✓	✓	✓	!
Konstantinova et al., 1995	!	✓	✓	✓	✓	✓	✓	!
Konstantinova et al., 1997	!	✓	✓	✓	✓	✓	✓	!
Konstantinova, 1991	!	✓	✓	✓	✓	✓	✓	!
Kovardakov et al., 1976	!	✓	✓	✓	✓	✓	✓	!
Krieger et al., 2021	✓	✓	✓	✓	✓	✓	✓	✓
Kurbanov et al., 1977	!	✓	✓	✓	✓	✓	✓	!
Kuzichkin et al., 2022	✓	✓	✓	✓	✓	✓	✓	✓
Kuzichkin et al., 2023	✓	✓	✓	✓	✓	✓	✓	✓
Larkin et al., 1972	!	✓	✓	✓	✓	✓	✓	!
Lesniak et al., 1998	!	✓	✓	✓	✓	✓	✓	!
Lesniak et al., 1999	!	✓	✓	✓	✓	✓	✓	!
Li et al., 2022	!	✓	✓	✓	✓	✓	✓	!
Lund and Dowdle, 1977	!	✓	✓	✓	✓	✓	✓	!
Manie et al., 1991	!	!	✓	✓	✓	✓	✓	!
Mehta et al., 2000	!	✓	✓	✓	✓	✓	✓	!
Mehta et al., 2022	!	✓	✓	✓	✓	✓	✓	!
Mehta et al., 2024	!	✓	✓	✓	✓	✓	✓	!

Study	Bias due to confounding	Bias in selection of participants into the study	Bias in classification of interventions	Bias due to deviations from intended interventions	Bias due to missing data	Bias in measurement of outcomes	Bias in selection of the reported result
Meshkov et al., 1995	!	✓	✓	✓	✓	✓	✓
Meshkov et al., 1998	!	✓	✓	✓	✓	✓	✓
Mishra et al., 2010	!	✓	✓	✓	✓	✓	✓
Mishra et al., 2011	!	✓	✓	✓	✓	✓	✓
Mishra et al., 2012	!	✓	✓	✓	✓	✓	✓
Moraes et al., 2023	✓	✓	✓	✓	✓	✓	✓
Moraes et al., 2024	✓	✓	✓	✓	✓	✓	✓
Morukov et al., 2010	!	✓	✓	✓	✓	✓	✓
Morukov et al., 2011	!	✓	✓	✓	!	✓	✓
Morukov et al., 2013	!	✓	✓	✓	✓	✓	✓
Mrakic-Sposta et al., 2022	!	✓	✓	✓	✓	✓	✓
Muchmore and Shurley, 1974	!	✓	✓	✓	✓	✓	✓
Muchmore et al., 1970	!	✓	✓	✓	✓	✓	✓
Muller et al., 1995	!	✓	✓	!	✓	✓	✓
Niemann et al., 2020	✗	✓	✓	✓	✓	✓	✓
Nikolaeva et al., 1982	!	✓	✓	✓	✓	✓	✓
Novikov et al. 1991	!	✓	✓	✓	✓	✓	✓
Novoderzhkina et al., 1996	!	✓	✓	✓	✓	✓	✓
Nwanaji-Enwerem et al., 2020	✓	✓	✓	✓	✓	✓	✓
Pastushkova et al., 2021	✓	✓	✓	✓	✓	✓	✓
Poliakov et al., 2005	✗	✓	✓	✓	✓	✓	✓
Ponomarev et al., 2015	!	✓	✓	✓	✓	✓	✓
Roberts-Thomson et al., 1985	!	✓	✓	✓	✓	✓	✓
Ryabinin et al., 1972	!	✓	✓	✓	✓	✓	✓
Rykova et al., 2004	!	✓	✓	✓	✓	✓	✓
Rykova et al., 2006	!	✓	✓	✓	✓	✓	✓
Rykova et al., 2008	!	✓	✓	✓	✓	✓	✓
Sakai et al., 2004	✓	✓	✓	✓	✓	✓	✓
Sapov et al., 1981	!	✓	✓	✓	✓	✓	✓
Schmitt et al., 1995	!	✓	✓	✓	✓	✓	✓
Schmitt et al., 1996	!	✓	✓	✓	✓	✓	✓
Schmitt et al., 2000	!	✓	✓	✓	✓	✓	✓
Shearer et al., 2001	!	✓	✓	✓	✓	✓	✓
Shearer et al., 2002	!	✓	✓	✓	✓	✓	✓
Shearer et al., 2009	!	✓	✓	✓	✓	✓	✓
Shirai et al., 2003	!	✓	✓	✓	✓	✓	✓
Sonnenfeld et al., 1992	✗	✓	✓	✓	✓	✓	✓
Sonnenfeld et al., 2007	!	✓	✓	✓	✓	✓	✓
Spielmann et al., 2018	✓	✓	✓	✓	✓	✓	✓
Spielmann et al., 2019	✓	✓	✓	✓	✓	✓	✓
Stahn et al., 2017	✓	✓	✓	✓	✓	✓	✓
Stowe et al., 2011	!	✓	✓	✓	✓	✓	✓
Strewe et al., 2015	!	✓	✓	✓	✓	✓	✓
Strewe et al., 2019	✓	✓	✓	✓	✓	✓	✓
Tashpulatov et al., 1971	!	✓	✓	✓	✓	✓	✓
Tashpulatov et al., 1976	!	✓	✓	✓	✓	✓	✓
Tingate et al., 1997	!	✓	✓	✓	✓	✓	✓
Trudel et al., 2009	✓	✓	✓	✓	✓	✓	✓
Uchakin et al., 2002	!	✓	✓	✓	✓	✓	✓
Uchakin et al., 2006	!	✓	✓	✓	✓	✓	!
Uchakin et al., 2007	!	✓	✓	✓	✓	✓	✓
Volozhin et al., 1999	!	✓	✓	✓	✓	✓	✓
Volozhin et al., 2001	!	✓	✓	✓	✓	✓	✓
Vorob´ev et al., 1984	!	✓	✓	✓	✓	✓	✓
Vorob´ev et al., 1986	!	✓	✓	✓	✓	✓	✓
Vorobyov et al., 1983	!	✓	✓	✓	✓	✓	✓
Walford et al., 1992	!	✓	✓	✓	✓	✓	✓
Xu et al., 2013	!	✓	✓	✓	✓	!	✓
Xu et al., 2016	!	✓	✓	✓	✓	!	✓
Xun et al., 2018	!	✓	✓	✓	✓	✓	✓
Yadav et al. 2012	!	✓	✓	✓	✓	✓	✓
Yi et al., 2014	✓	✓	✓	✓	✓	✓	✓
Yi et al., 2015	!	✓	✓	✓	✓	✓	✓
Yuan et al., 2019	!	✓	✓	✓	✓	✓	✓
Zakovska et al., 2023	!	✓	✓	✓	✓	✓	✓

D1 Bias due to confounding
D2 Bias in selection of participants into the study
D3 Bias in classification of interventions
D4 Bias due to deviations from intended interventions
D5 Bias due to missing data
D6 Bias in measurement of outcomes
D7 Bias in selection of the reported result

✓ **Low risk**
! **Moderate risk**
✗ **Serious risk**
